# Harnessing Explainable AI to Explore Structure–Activity Relationships in Artificial Olfaction

**DOI:** 10.1021/acsami.5c13990

**Published:** 2025-09-08

**Authors:** Yota Fukui, Kosuke Minami, Genki Yoshikawa, Koji Tsuda, Ryo Tamura

**Affiliations:** † Graduate School of Frontier Sciences, 13143 The University of Tokyo, 5-1-5 Kashiwanoha, Kashiwa, Chiba 277-8568, Japan; ‡ Center for Basic Research on Materials, 242079 National Institute for Materials Science, 1-1 Namiki, Tsukuba, Ibaraki 305-0044, Japan; § Research Center for Macromolecules and Biomaterials, National Institute for Materials Science, 1-1 Namiki, Tsukuba, Ibaraki 305-0044, Japan; ∥ Materials Science and Engineering, Graduate School of Pure and Applied Science, University of Tsukuba, 1-1-1 Tennodai, Tsukuba, Ibaraki 305-8571, Japan

**Keywords:** chemical sensor arrays, artificial olfaction, receptor materials, explainable AI, score-CAM, convolutional neural network

## Abstract

Chemical sensor arrays mimic the mammalian olfactory system to achieve artificial olfaction, and receptor materials resembling olfactory receptors are being actively developed. To realize practical artificial olfaction, it is essential to provide guidelines for developing effective receptor materials based on the structure–activity relationship. In this study, we demonstrated the visualization of the relationship between sensing signal features and odorant molecular features using an explainable AI (XAI) technique. We focused on classification tasks and employed a convolutional neural network (CNN) and score-class activation mapping (Score-CAM) methods. The results obtained from analyzing the 94 odor samples prepared using pure solvents indicate that the information regarding the active receptor materials and data points in the signals and the structure–activity relationship could be accurately extracted. Therefore, using XAI techniques to analyze sensor signals from odor data is an important technique for advancing artificial olfaction.

## Graphical abstract

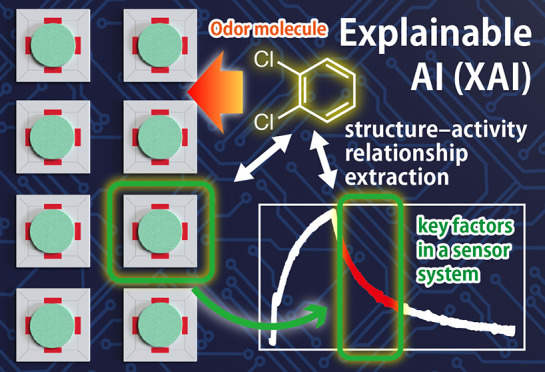

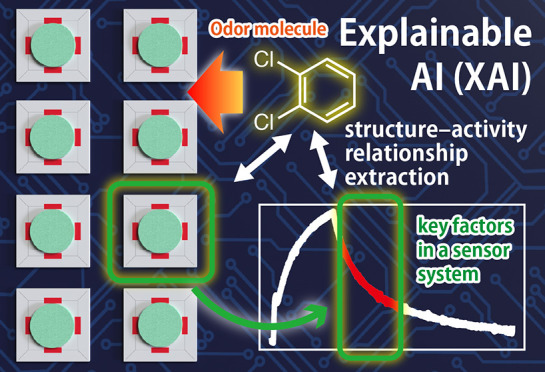

## 1 Introduction

The potential of odors has attracted considerable attention in various fields, including food, agriculture, the environment, cosmetics, healthcare, and medicine. Olfactory information is carried by a wide variety of odorant molecules. An odor often consists of a complex mixture of hundreds of different molecules. It is estimated that more than 400,000 different compounds are odorous to the human nose.^1^ The mammalian olfactory system detects and discriminates such complex odors through a highly organized process. A key aspect of this system is the spatial mapping of odorant molecular features within the olfactory bulb.^2^ Understanding the mapping of odorant molecular features in the mammalian olfactory bulb based on the structure–activity relationship between odorants and olfactory receptors enhances our knowledge of sensory processing and our perception.

Chemical sensor arrays that mimic the mammalian olfactory system have been developed to detect, discriminate, and identify target analytes to achieve artificial olfaction.^3–10^ In the development of artificial olfaction, signal processing is of great importance to detect and discriminate odors. By collecting various sensor responses from different odor samples, data-driven analysis can be performed to predict the categories of unknown odors and extract quantitative information about target odors.^11–16^ Most current approaches, including machine learning techniques, focus on identifying odors based on empirically extracted features from signal responses rather than understanding the structure–activity relationship between signal features and odorant molecular features.^17^ In contrast, the social implementation of artificial olfaction requires the development of receptor materials capable of covering a wide range of target odors. To guide the development of such receptor materials, it is essential to identify active receptor materials depending on the odorant molecules and to extract important signal features based on data-driven analysis rather than empirical intuition.

In this study, we demonstrated visualization of the relationship between signal features and odorant molecular features using an explainable AI (XAI) technique (Figure 1). Recently, XAI techniques for understanding, not just using AI for prediction and pattern recognition as a black box, are attracting attention. Class activation mapping (CAM)^18^ is a technique for identifying important factors in classification for convolutional neural network (CNN) models and is well-known as an XAI method in image recognition. The main advantage of using XAI is its ability to extract active important factors for each individual sample, that is, the structure–activity relationship. This enables the extraction of more detailed information than the importance values provided by decision-tree-based models such as random forest (RF), offering a guiding principle for the development of receptor materials. Although the most common CAM algorithm is Grad-CAM,^18,19^ it is a gradient-based algorithm and suffers from the vanishing gradient problem. This issue causes the activation mapping to contain significant noise. To address this, gradient-free CAM algorithms such as Eigen-CAM,^20^ Ablation-CAM,^21^ and Score-CAM^22^ have been developed. Among these, we employed Score-CAM to stably analyze the signal responses of artificial olfaction. To prepare the original data set for the analysis using Score-CAM, 94 vapors of pure solvents were measured with 14 channels of nanomechanical membrane-type surface stress sensors (MSSs),^23,24^ which transduce the surface stress resulting from the physicochemical interaction between receptor materials and odorant molecules into electrical signals. The measured vapor data are published in an open-access data repository (NIMS Materials Data Repository, 10.48505/nims.5556). By applying Score-CAM for the results of the CNN, we successfully visualized not only the active features of receptor materials for classifying categories but also the structure–activity relationship between signal features and odorant molecular features, e.g., the existence of oxygen atoms (which are related to the functional groups) and ring structures. The present approach not only provides a guideline for developing effective receptor materials based on the structure–activity relationship but also has the potential to link artificial olfaction to our perception.

**Figure 1:**
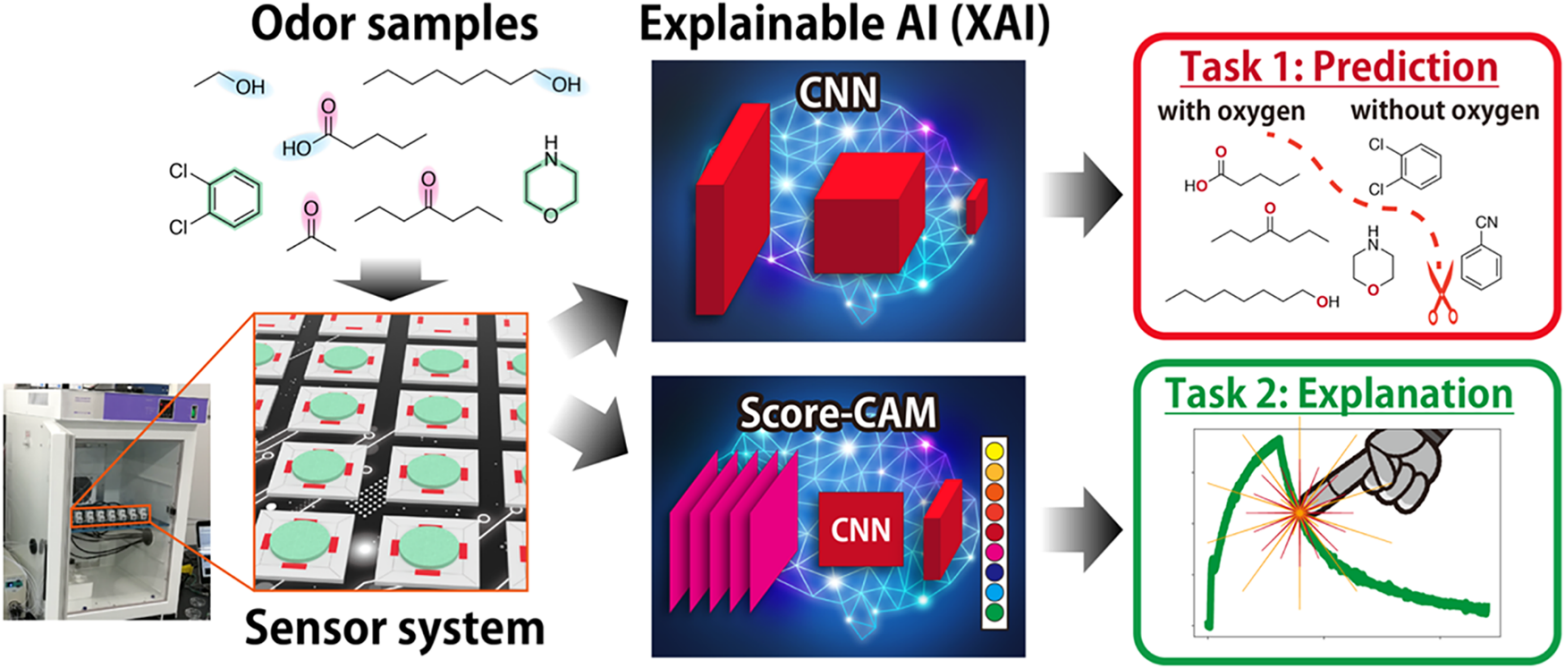
Flowchart of our signal analysis based on XAI for predicting the category of samples and understanding the important parts of the signals.

## 2 Materials and Methods

### 2.1 Odor Samples

In this study, we used 94 different pure solvents as odor samples, as summarized in Table S1. All of the solvents were purchased from Sigma-Aldrich, Tokyo Chemical Industry, and FUJIFILM Wako Pure Chemical Corporation. For each solvent, three types of odor samples were prepared with varying concentrations (5, 10, and 20%) diluted with pure nitrogen.

### 2.2 Sensing Procedure

In this study, an MSS was employed as an electronic nose. The fabrication of MSS chips and their operational principles have been previously documented,^25,26^ and several studies have reported applications for odor analysis by integrating machine learning with an MSS.^10,14,15,27–29^ The MSS chips were purchased from NanoWorld AG (Switzerland). Each channel of the MSS chips was coated with polymers and silica–titania hybrid nanoparticles (STNPs)^30^ dissolved in *N*,*N*-dimethylformamide (FUJIFILM Wako Pure Chemical Corporation) using an inkjet spotter (LaboJet-500SP, Microjet Corporation, Japan) equipped with a nozzle (IJHBS-300, Microjet Corporation, Japan). The detailed coating procedure has been previously summarized by Xu et al.^28^ The 14 types of receptor materials used in this study are listed in Table 1. Channels 8 and 9 were coated with the same receptor materials; however, the coating methods differed.^28^ The odor sample was introduced into each channel of the MSS for 30 s, called the sampling process, when odorous molecules adsorb on a receptor layer (adsorption process), followed by the introduction of pure nitrogen for 90 s, called the purging process, when odorous molecules desorb from a receptor layer (desorption process).

**Table 1:** Receptor Materials in the MSS

channel	material
1	polystyrene
2	poly(4-methylstyrene)
3	poly(4-vinylphenol)
4	polymethylmethacrylate
5	polycaprolactone
6	poly(vinylidene fluoride)
7	tenaxTA 20–35
8	tenaxTA 60–80
9	tenaxTA 60–80
10	aminopropyl STNPs
11	octadecyl STNPs
12	phenyl STNPs
13	glycidyl STNPs
14	SiO_2_C_16_TAC

### 2.3 Machine Learning

In this study, a CNN model was used as the machine learning model to perform the classification task. The structure of the CNN is shown in Figure 2 and is based on previous studies^31–33^ that achieved high recognition accuracy. The hyperparameters within the CNN, such as epochs, were determined to enhance prediction accuracies. The parameters used in the CNN are summarized in the Python code implemented with TensorFlow,^34^ provided as Code 1 in the Supporting Information. The input data for the CNN model were prepared by using sensor signals recorded at three different concentrations. Sensor signals were sampled at 20 Hz for 120 s, from which data points were extracted every 3 s, resulting in 40 data points for each channel. Using 14 channels with different receptor materials, the size of the input matrix was 40 × 14 × 3. The signal value at 0 s was set to zero, and the signals were normalized such that the maximum value of the signals of the three concentrations for each channel was 1.

**Figure 2:**
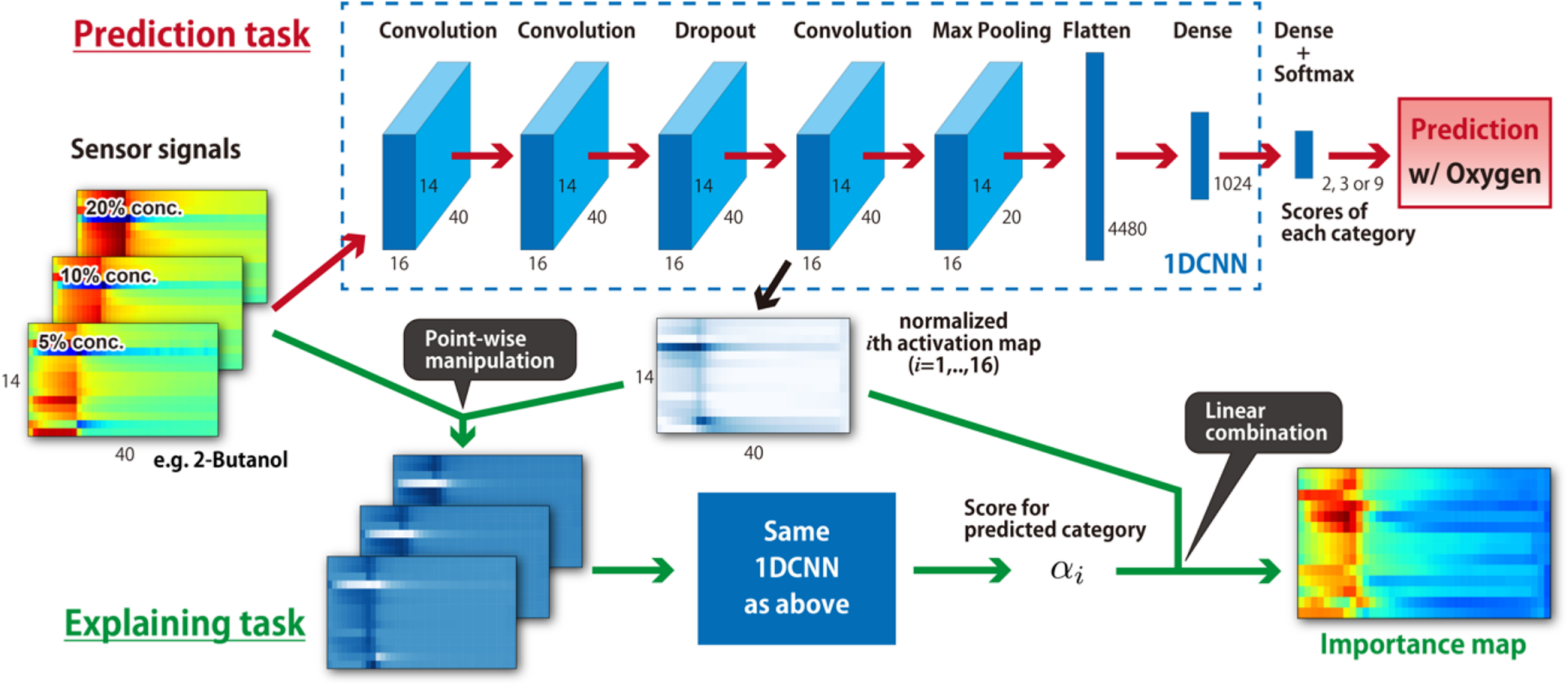
Structure of the CNN used for predicting categories and the process flow of the Score-CAM method used for visualizing the important parts of the descriptors.

The Score-CAM method, which was used to explain the task (Figure 2), generally uses the results from the final convolution layer in the trained CNN. In our CNN structures, 16 activation maps were obtained in the final convolution layer, where each map was normalized using min–max normalization. Using each activation map, masked input data were generated through point-wise manipulation of the input data, and the classification results were obtained for the masked data set using the trained CNN. This procedure was performed for all 16 activation maps, and the scores of the predicted categories were evaluated. Using a linear combination of the evaluated scores, the 16 activation maps were combined, forming an importance map for the input signals. The process for conducting Score-CAM is summarized in Figure 2.

## 3 Results

Using the MSS, in which 14 channels were coated with receptor materials, as listed in Table 1, 94 different vapors with three different concentrations were measured (see also Section 2). The CNN was employed to categorize different types of molecules and classify molecules with or without oxygen atoms as well as those with or without ring structures. The categories of the odor samples (acids, alcohols, aliphatic hydrocarbons, aromatic hydrocarbons, esters, ethers, halogenated compounds, ketones, and others) are given in Table S1. The prediction accuracies and macro F1-scores obtained from leave-one-out cross-validation are summarized in Figure 3. For comparison with conventional methods, we also employed support vector machine (SVM) and RF models. For SVM and RF, conventional four-dimensional features for each channel were generated by focusing on signal responses that may indicate chemical/physicochemical interactions, such as adsorption and desorption processes (see Supporting Note A).^14^ Thus, 168-dimensional (= 4 (dimension of signal features) × 14 (number of channels) × 3 (different concentrations)) features are used to train SVM and RF. To train the SVM and RF models, the scikit-learn package^35^ was utilized. Although hyperparameters were tuned by using GridSearchCV, the prediction accuracy did not differ significantly from that obtained with the default settings in scikit-learn. Therefore, in this study, we showed the results using the default settings of the scikit-learn package for both SVM and RF. The prediction performance of the CNN in terms of accuracy and macro F1-score for the three classification tasks (i.e., category, presence of oxygen atoms, and presence of ring structures) was either superior to or comparable with that of conventional machine learning models. The prediction accuracies of the CNN for the three tasks exceeded 0.8, with 0.830 for category classification, 0.883 for oxygen atoms classification, and 0.851 for ring structures classification (Figure 3a), indicating that the CNN has sufficient prediction accuracy. The macro F1-scores for each category obtained using CNN are shown in Figure 3c. Detailed prediction results are given in Table S1, and the confusion matrices for each classification task are shown in Figure 4. The CNN demonstrated a clear advantage over SVM and RF in terms of true positive rates across all classification tasks. This superiority is particularly evident in the prediction of the ethers category in the category classification task, where both SVM and RF failed entirely, achieving a true positive rate of 0%. In contrast, the CNN successfully identified 50% of the samples in this category, highlighting its enhanced capability in capturing complex patterns of signals that conventional methods could not detect.

**Figure 3:**
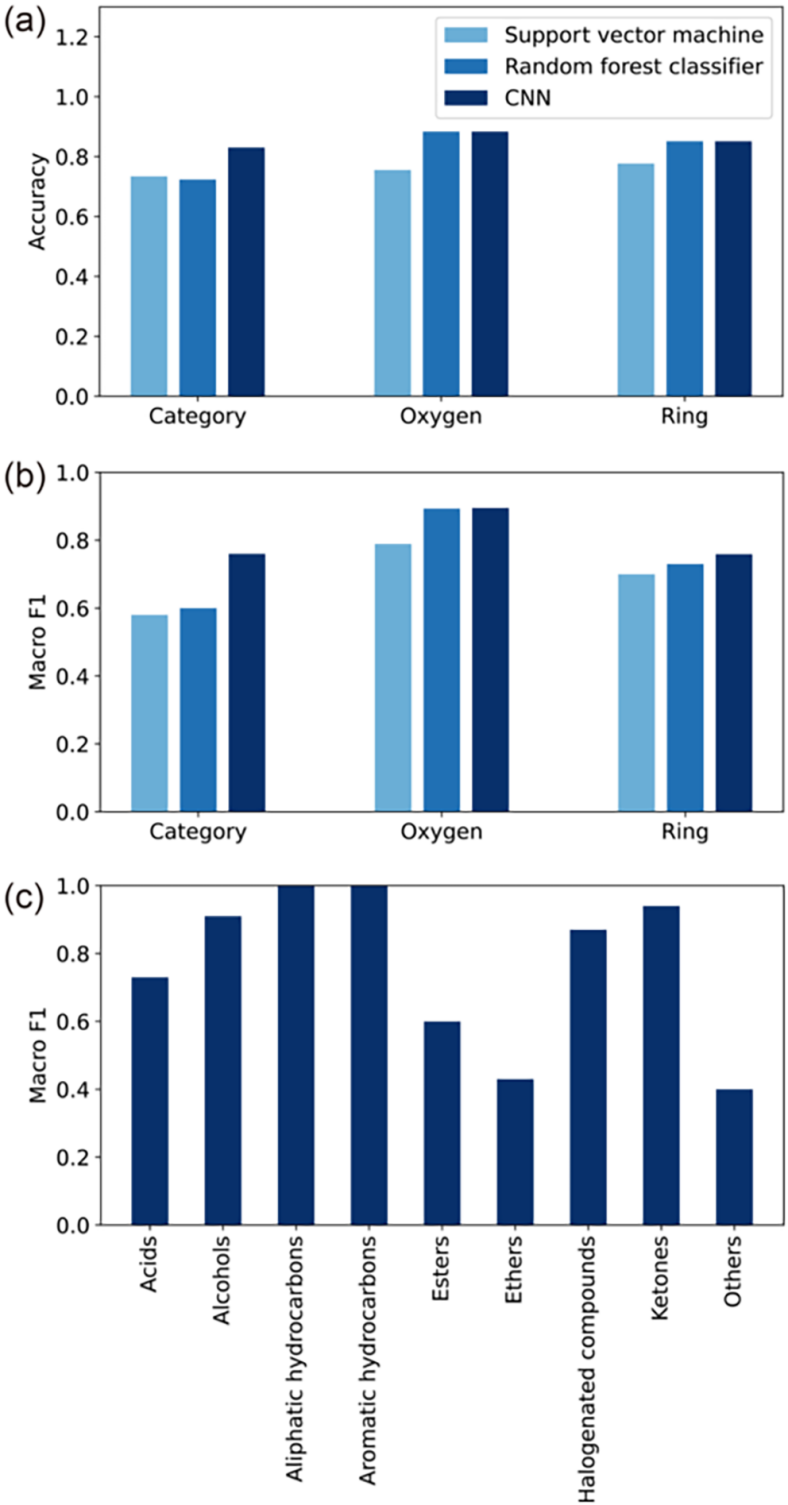
(a) Accuracy and (b) macro F1-scores obtained from leave-one-out cross-validation using the CNN model compared with conventional methods (support vector machine and random forest classifier) for three classification tasks: nine categories of molecule types, with or without oxygen atoms, and with or without ring structures. (c) Macro F1-scores for each category in the nine-category classification task.

**Figure 4:**
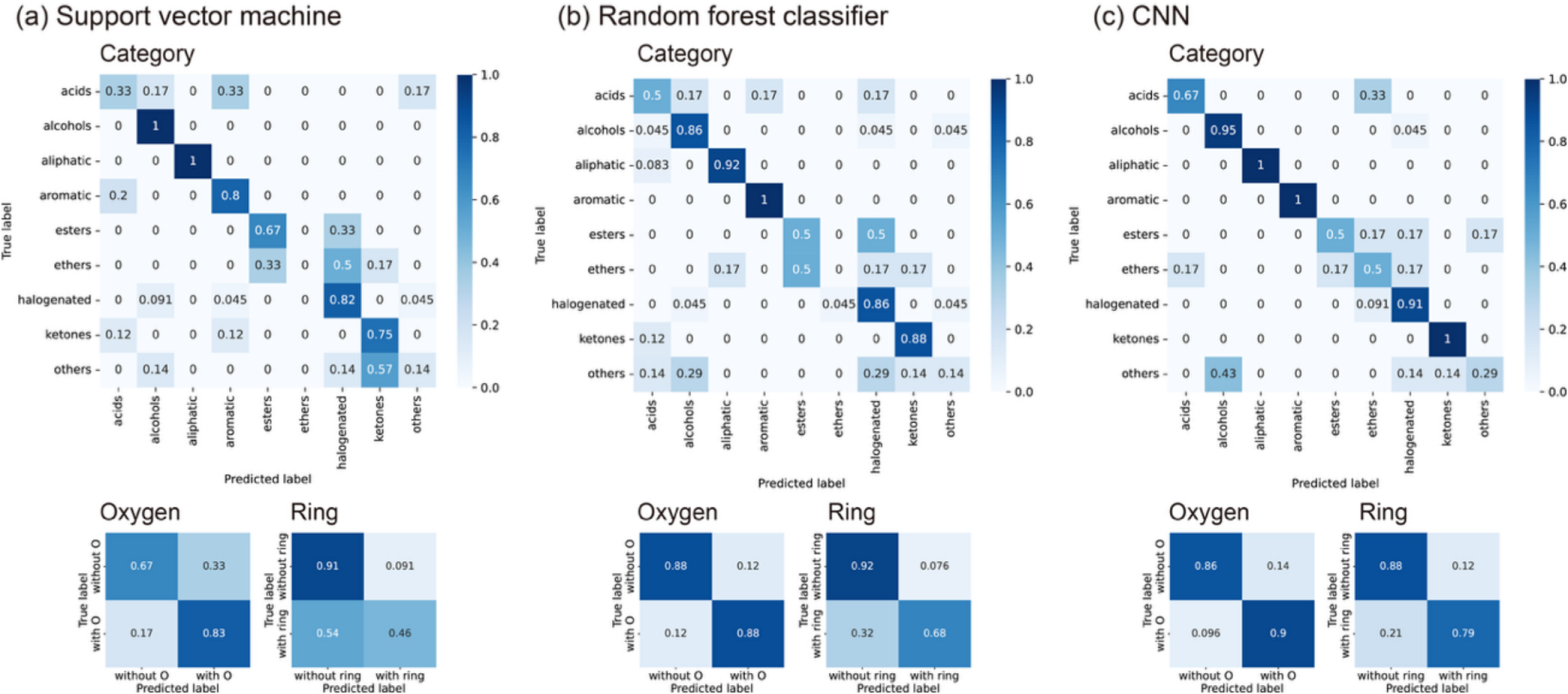
Confusion matrices for three classification tasks: nine categories of molecule types, with or without oxygen atoms, and with or without ring structures using the (a) support vector machine, (b) random forest classifier, and (c) CNN.

The active receptor materials with their corresponding active data points in the signal outputs for each classification task were visualized as importance maps by applying Score-CAM. These maps were obtained for each sample after the category of the sample was predicted (see Figure 2). The importance maps for all of the samples are summarized in Figure 5, based on a leave-one-out cross-validation scheme, and each map is shown in Figure S1. Overall, results indicate that the importance of the sampling process in 1–30 s is larger than 30–120 s for the category prediction. For categories with higher prediction performance—namely, alcohols, aliphatic hydrocarbons, aromatic hydrocarbons, halogenated compounds, and ketones—a clear similarity in the importance maps is observed within each category. In contrast, for categories with lower prediction performance—such as acids, esters, ethers, and others—the active regions vary significantly across samples. This result indicates that the current sensor array lacks sufficient receptor materials for accurately identifying these latter categories, and that the development of new receptor materials is necessary to improve prediction performance. To integrate these results, the average importance maps were plotted using the average of the samples with successful category predictions. Figure 6 shows the importance maps for the classification of the nine categories. The active data points and channels differed depending on the category. For example, a wide range of channels among 14 channels were used to classify alcohols and aliphatic hydrocarbons, whereas only channel 3 was mainly used for esters classification. For the acids classification, the data point at around 25 s on channel 14 (i.e., adsorption process) was active, while the active data points for the classification of halogenated compounds were around 20 s on channel 4 and after 30 s on channel 13 (i.e., desorption process).

**Figure 5:**
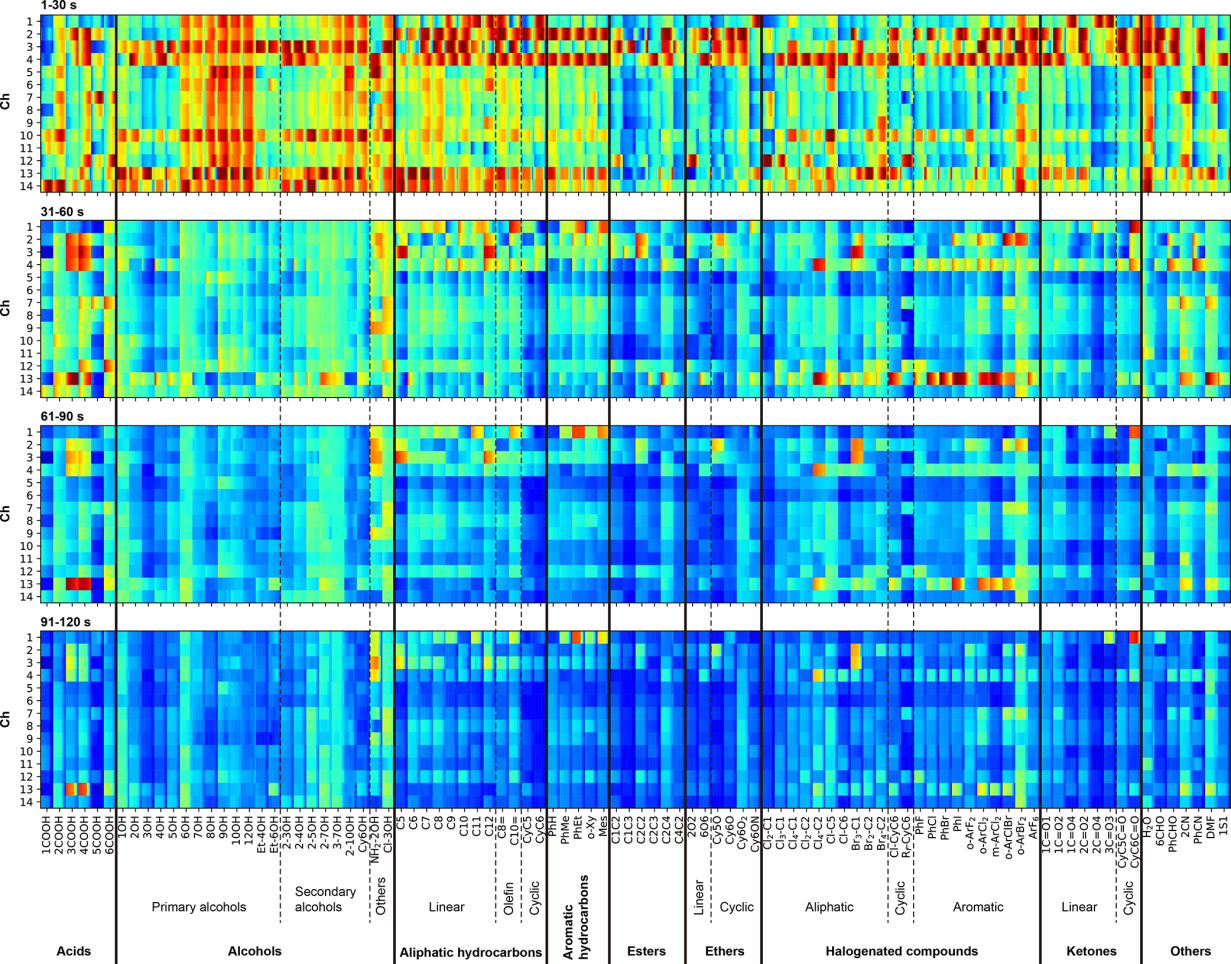
Importance maps were generated using the Score-CAM method when the category is predicted.

**Figure 6:**
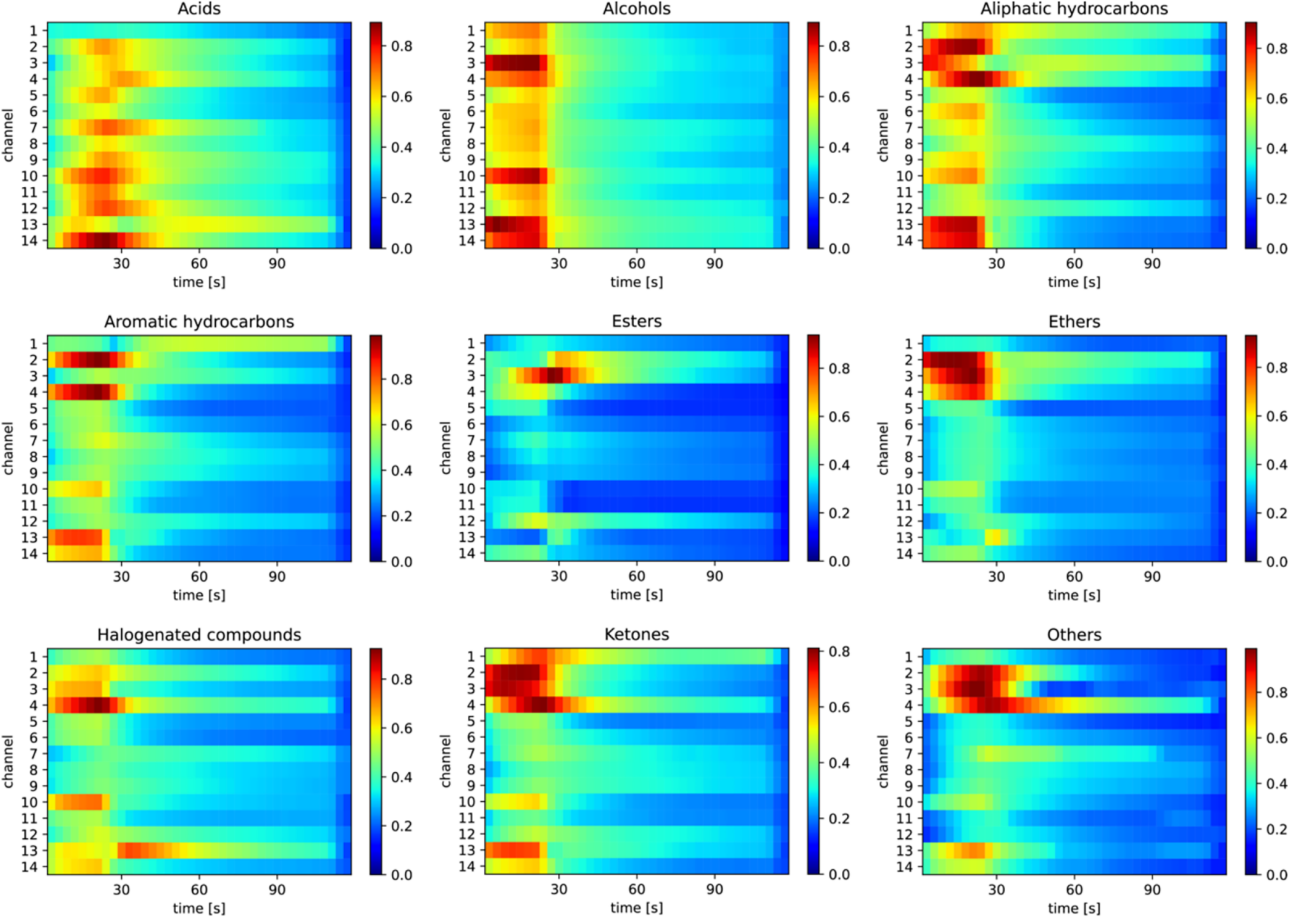
Averaged importance maps generated using the Score-CAM method based on the prediction of nine-category targets.

The importance maps for the classification of oxygen atoms and ring structures are shown in Figure 7, and for each sample, the importance maps are summarized in Figures S2–S5. For the classification of the presence of oxygen atoms, most channels in the adsorption process (until 30 s) and at the beginning of the desorption process (i.e., just after 30 s) were active, whereas only a limited number of channels were used for the classification of the presence of ring structures.

**Figure 7:**
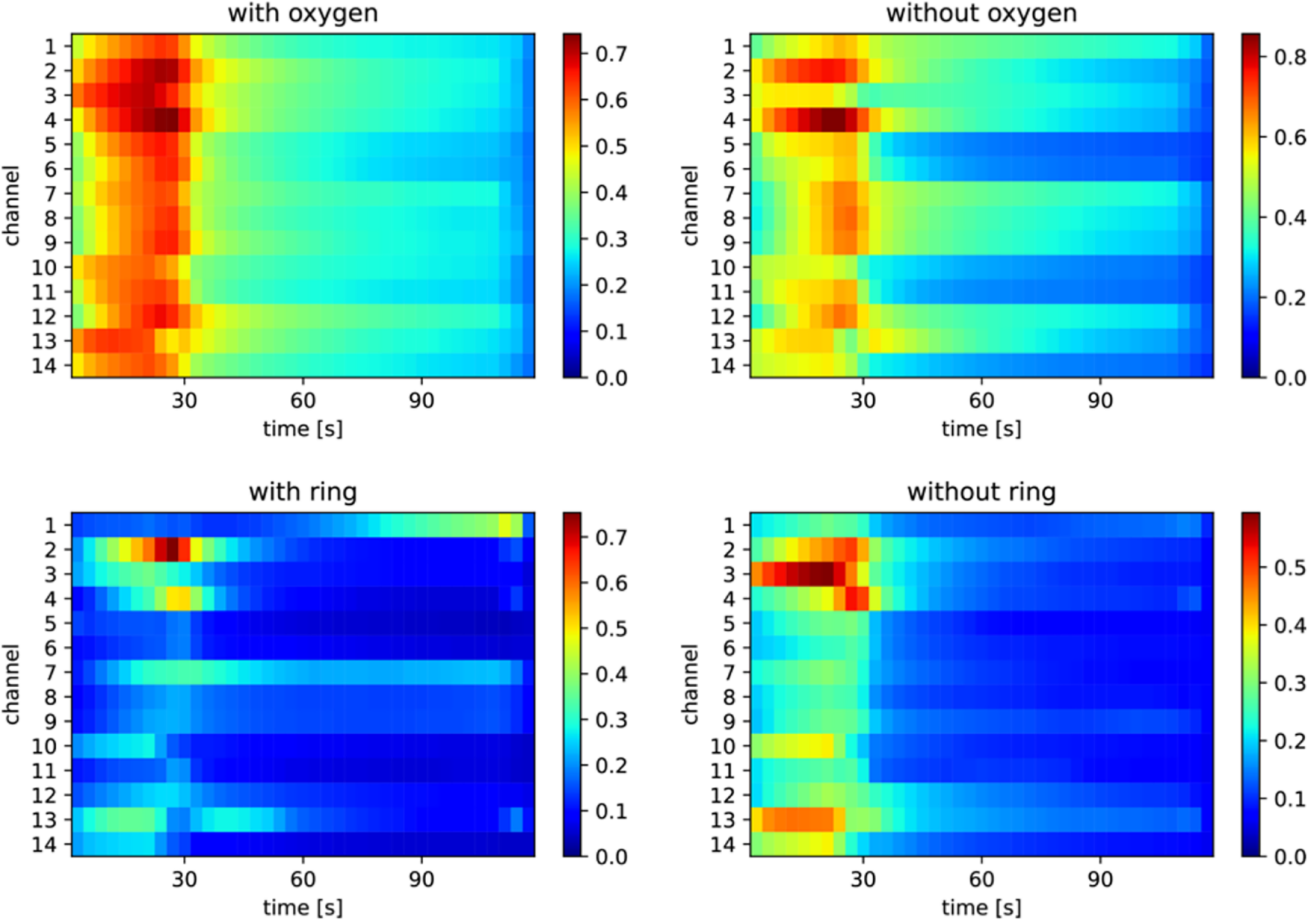
Averaged importance maps with successful category predictions generated by using the Score-CAM method for oxygen and ring structure classifications.

We compared the importance maps obtained by Score-CAM with the feature importance values derived from the RF model. In the RF model, 168 features were used. The feature importances were averaged across the four-dimensional signal features and three concentration levels to yield the overall channel importances, as shown in Figure 8. For category classification, Score-CAM analysis identified channels 2, 3, and 4 as active across all targets (Figure 6). This finding is consistent with the RF model results (Figure 8a). Similarly, for the classification of vapors with or without oxygen atoms, channels 2, 3, and 4 were active in the Score-CAM maps. For ring structure classification, channels 2, 3, 4, and 13 were active. With a few exceptions, these are consistent with the results of RF importance. Moreover, both Score-CAM and the RF model indicate that channels 6, 7, 8, and 9 are not important. The consistency between the Score-CAM and RF results suggests that active channels can be appropriately identified using Score-CAM.

**Figure 8:**
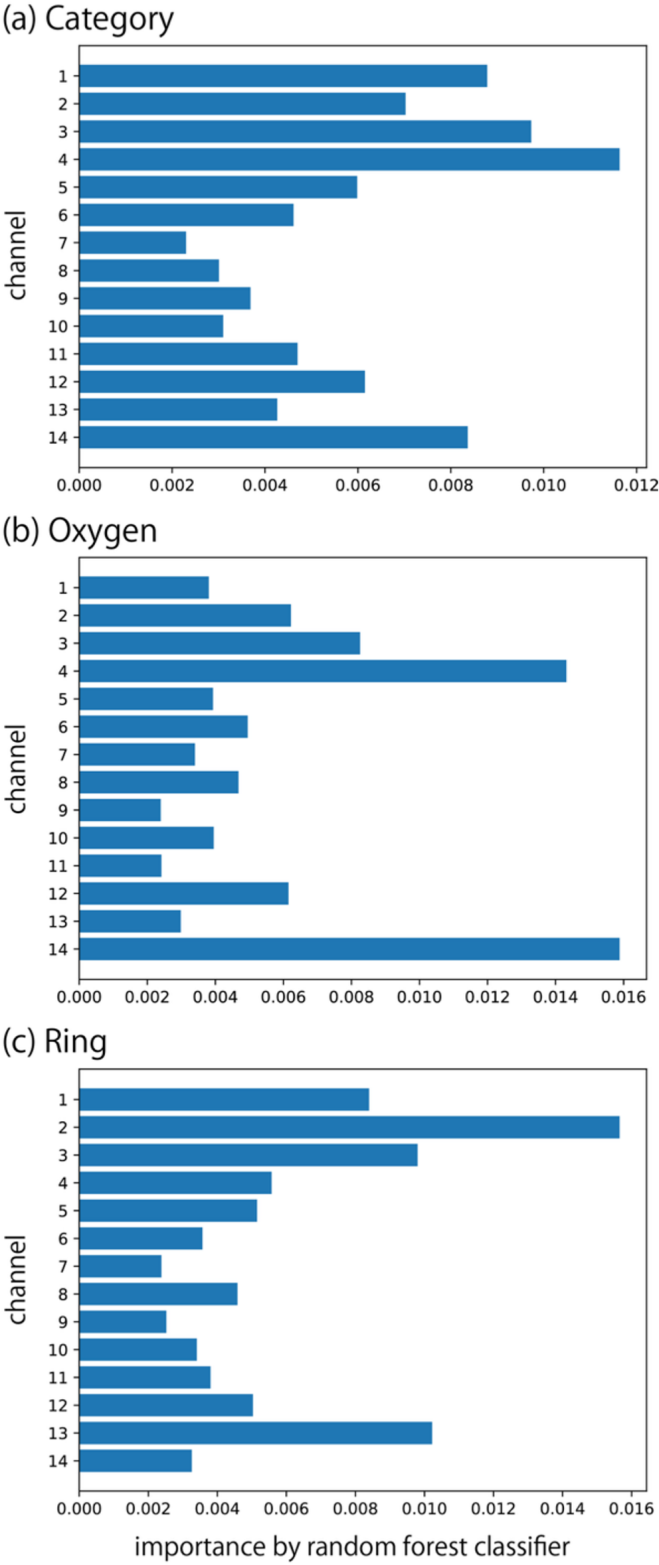
Feature importance by RF models for three classification tasks: (a) nine categories of molecule types, (b) with or without oxygen atoms, and (c) with or without ring structures.

## 4 Discussion

CAM methods, which are a type of XAI technique specialized for CNNs in image recognition, visualize what CNNs focus on in an image. CNNs are powerful tools that have been used for sensor signal analysis in electronic noses.^32,33,36–38^ Thus, by treating the electric signals obtained from electronic noses as an image using a CNN, we applied Score-CAM to visualize where a CNN detects the signals for classification. Here, we discuss the results based on chemical considerations.

To demonstrate the Score-CAM-based visualization, we first focused on predicting categories classified by functional groups of the measured odor samples. Since the signal responses of chemical sensors, including nanomechanical sensors, are obtained from chemical/physicochemical interactions between the target molecules and receptor materials,^10,39–43^ they are expected to classify the categories of molecules that align with their chemical properties. As expected, the classification accuracy of the CNN exceeded 0.8, which was higher than or comparable to that of conventional methods (i.e., SVM and RF) as shown in Figure 3a. By applying the Score-CAM method, the active receptors and data points were clearly visualized as can be seen in Figure 5. The active receptors and data points were highly dependent on the categories (see Figure 6). For example, for the prediction of alcohols, the adsorption process is active because of their fast adsorption and diffusion into the receptor layer.^14,44^ In contrast, the purge process is also important for acid predictions. Since acids tend to remain in the receptor materials, possibly owing to ionization in the receptor layer,^45^ such slow desorption is reflected in the importance map. Notably, the classification accuracy of the ethers was rather low at about 0.5, and the worst classification accuracy of around 0.4 was obtained for the others category, which includes water, aldehydes, sulfur-containing compounds (i.e., dimethyl sulfide), and nitrogen-containing compounds (i.e., *N,N*-dimethylformamide, acetonitrile, and benzonitrile). The ethers category in this study consisted of water-miscible cyclic ether, tetrahydrofuran, water-immiscible cyclic ether, tetrahydropyran, water-immiscible less polar ether, diethyl ether, water-immiscible less polar ether with long alkyl chains, and dihexyl ether. Therefore, each ether may not align with a single chemical property of ethers category because a leave-one-out cross-validation was used for calculating the accuracy of the CNN model, resulting in low accuracy similar to that of the others category. As shown in Figure 5, the active regions vary significantly across samples in the ethers category. These findings indicate that the development of receptor materials specifically responsive to ether compounds is essential for improving identification performance, and this will be an important direction for future work.

In addition to classifying the chemical properties of samples in terms of category classification, we then demonstrated a rather difficult task: classifying the presence or absence of oxygen atoms. Heteroatoms in organic compounds, such as oxygen, nitrogen, sulfur, and halogens, result in chemical properties different from those of simple hydrocarbons. These different chemical properties lead to distinct dynamic responses of the chemical sensors. Among these heteroatoms, we also classified the presence of oxygen atoms using CNN because a large number of samples contain oxygen atoms in 94 samples. The prediction results (Table S1) indicate that all the nitrile compounds (i.e., acetonitrile and benzonitrile) and some of the halogenated aliphatic compounds used in this study were incorrectly classified as having oxygen atoms. Since nanomechanical sensors detect general physicochemical interactions (e.g., hydrogen bonding or van der Waals forces) rather than specifically targeting oxygen-containing groups, the high polarity and hydrogen bonding nature of cyano groups in nitriles could result in signals similar to those of carbonyl or hydroxyl groups, leading to misclassification. Conversely, all of the halogenated aromatic compounds and chlorinated compounds were correctly classified, while the highly fluorinated compound (i.e., perfluoromethylcyclohexane), which shows hydrophobic and lipophobic properties, was misclassified.

Since the presence or absence of oxygen atoms can be predicted with high accuracy, we checked whether differences in the number of oxygen atoms in the molecules could also be correctly classified. The molecules in the data set contained 0, 1, or 2 oxygen atoms. A classification model for these three categories was trained using a CNN with an accuracy of 0.798 (Table S1). This enhanced prediction performance indicates that MSS correctly captures the distinction between the functional groups, such as hydroxyl, carboxy, and ketone groups. On the other hand, some molecules containing heteroatoms other than oxygen—such as ethanolamine, dichloromethane, bromoform, acetonitrile, and dimethyl sulfide—are misclassified in both the oxygen atom count classification and the odor category classification. We consider this to be due to the influence of heteroatoms on the classification process. Increasing the number of samples containing various heteroatoms and addressing the effects of existing heteroatoms represent important directions for future work.

In so-called static-mode nanomechanical sensing, signals are given by the volume expansion of receptor materials derived from the adsorption/desorption of molecules.^9,10,46,47^ Accordingly, in principle, the molecular structure of the odor sample affects the shape of signals.^10,48^ Therefore, in addition to oxygen atom classification, the more challenging task of classifying the presence or absence of ring structures was attempted. Notably, in this classification, not only aliphatic cyclic structures, including 5-membered ring (e.g., cyclopentane) and 6-membered rings (e.g., cyclohexanone), but also aromatic rings (i.e., benzene ring) and heterocyclic structures (e.g., tetrahydrofuran and morpholine) are assigned as the ring structure (Table S1). Thus, the group of compounds treated with ring structures does not match the chemical properties, making classification by nanomechanical sensors more difficult. The prediction accuracy of the CNN exceeded 0.8, indicating that ring structures can be classified by a CNN with a relatively high accuracy. Significantly, all of the aromatic compounds were correctly classified by the CNN. According to the results of the Score-CAM (Figure 7), only a few channels are active in the importance maps; however, poly­(4-methylstyrene) exhibits significant activity. This suggests that the correct classification of aromatic compounds may be attributed to the π–π interaction between the aromatic rings of poly­(4-methylstyrene) and those of the target molecules, while the accuracy of alicyclic compounds decreased because they include heteroatom-contained cyclic structures, various physicochemical interactions resulting from ring structures with carbonyl and hydroxyl groups, and structural differences between 5-membered and 6-membered rings.

## 5 Conclusions

We demonstrated the visualization of the relationship between sensing signal features and odorant molecule features in an electronic nose by utilizing an XAI technique. A deep learning method combined with CNN and Score-CAM methods was used to generate the importance map of the features. This method was applied to sensor data obtained using an MSS. The classification tasks included distinguishing between samples with or without oxygen atoms and with or without ring structures, in addition to categorizing them into nine different categories. The CNN model achieved prediction accuracies exceeding 0.8 for these classification problems. The importance map obtained using the Score-CAM method demonstrated that the active receptor materials and active data points were extracted for each classification problem. The XAI combining CNN and Score-CAM methods is a powerful tool for understanding the active receptor materials in electronic noses. The efficacy of receptor materials extracted by Score-CAM may vary depending on the target odor data sets and classification tasks. Therefore, applying XAI techniques to odor data sets containing a mixture of odors as well as to a more general odor data set, such as fruits and perfumes, would be an interesting and important direction for future work, which will interpret olfaction systems. Furthermore, XAI techniques not only extract active receptor materials but also aid in selecting an appropriate sensor system for the target classification problem of odors by analyzing data obtained from multiple sensor systems.

## Supplementary Material



## Data Availability

The data that support the findings of this study are available from the corresponding author, R.T., upon reasonable request.

## Notes

Python code for the definition of CNN model and training of CNN (Code 1); odor samples, category data, and all prediction results by CNN model (Table S1); importance maps for all the samples when the category classification was performed (Figure S1); importance maps generated using the Score-CAM method when the existence of oxygen atom is predicted (Figure S2); importance maps for all the samples when the oxygen atoms classification was performed (Figure S3); importance maps generated using the Score-CAM method when the existence of ring structure is predicted (Figure S4); importance maps for all the samples when the ring structures classification was performed (Figure S5); MSS signals of ethers at a concentration of 20% (Figure S6); definition of the four-dimensional features of the signals (Supporting Note A) (PDF)
