# Supplementary material for: Harnessing Explainable AI to Explore Structure–Activity Relationships in Artificial Olfaction
Source: ACS Appl Mater Interfaces. 2025 Sep 8;17(37):52728–37. doi: 10.1021/acsami.5c13990 (PMC12447402; doi:10.1021/acsami.5c13990)
Supplement: Supplementary file 1 [file am5c13990_si_001.pdf]

Supporting Information for

Harnessing Explainable AI to Explore Structure–Activity Relationships in Artificial Olfaction

Yota Fukui[1,2], Kosuke Minami[3,*], Genki Yoshikawa[3,4], Koji Tsuda[1,2], and Ryo Tamura[2,1,*]

[1]Graduate School of Frontier Sciences, The University of Tokyo, 5-1-5 Kashiwanoha, Kashiwa, Chiba 277-8568, Japan

[2]Center for Basic Research on Materials, National Institute for Materials Science, 1-1 Namiki, Tsukuba, Ibaraki 305-0044, Japan

[3]Research Center for Macromolecules and Biomaterials, National Institute for Materials Science, 1-1 Namiki, Tsukuba, Ibaraki 305-0044, Japan

[4]Materials Science and Engineering, Graduate School of Pure and Applied Science, University of Tsukuba, 1-1-1 Tennodai, Tsukuba, Ibaraki 305-8571, Japan

Email:
minami.kosuke@nims.go.jp (Kosuke Minami)
tamura.ryo@nims.go.jp (Ryo Tamura)

Code 1. Python code for the definition of CNN model and training of CNN using TensorFlow.

```
from tensorflow.keras import layers
from tensorflow.keras import models

def build_model():
    model.add(layers.Conv2D(filters = 16, kernel_size = (2, 1), strides = (1,1),
                padding = 'same', activation = 'relu', input_shape = (40, 14, 3))
    model.add(layers.Conv2D(filters = 16, kernel_size = (2, 1), strides = (1,1),
                padding = 'same', activation = 'relu'))
    model.add(layers.Dropout(rate = 0.2))
    model.add(layers.Conv2D(filters = 16, kernel_size = (2, 1), strides = (1,1),
                padding = 'same', activation = 'relu'))
    model.add(layers.MaxPooling2D(pool_size = (2,1), strides = (2,1),
                padding = 'same'))
    model.add(layers.Flatten())
    model.add(layers.Dense(units = 1024, activation = 'relu'))
    model.add(layers.Dense(units = 9, activation = 'softmax'))
    model.compile(optimizer = 'rmsprop', loss = 'categorical_crossentropy',
                metrics = ['acc'])
    return model

model = build_model()
model.fit(X, y, epochs = 100, batch_size = 1, verbose = 0)
```

Table S1. Odor samples, category data, and all prediction results by CNN model. With or without are denoted by "1" or "0". When the prediction is failed, "F" is shown.

Label	Samples	Category		Oxygen (w/ or w/o)		Ring (w/ or w/o)		No. of Oxygen	
		True	Prediction	True	Prediction	True	Prediction	True	Prediction
1COOH	Formic acid	Acids		1	1	0	0	2	2
2COOH	Acetic acid	Acids		1	1	0	0	2	2
3COOH	Propionic acid	Acids	Ethers (F)	1	1	0	1 (F)	2	1 (F)
4COOH	Butanoic acid	Acids		1	1	0	0	2	2
5COOH	Pentanoic acid	Acids	Ethers (F)	1	1	0	0	2	1 (F)
6COOH	Hexanoic acid	Acids		1	1	0	0	2	2
1OH	Methanol	Alcohols		1	0 (F)	0	0	1	0 (F)
2OH	Ethanol	Alcohols		1	1	0	0	1	1
3OH	1-Propanol	Alcohols		1	1	0	0	1	1
4OH	1-Butanol	Alcohols		1	1	0	0	1	1
5OH	1-Pentanol	Alcohols		1	1	0	0	1	1
6OH	1-Hexanol	Alcohols		1	1	0	0	1	1
7OH	1-Heptanol	Alcohols		1	1	0	0	1	1
8OH	1-Octanol	Alcohols		1	1	0	0	1	1
9OH	1-Nonanol	Alcohols		1	1	0	1 (F)	1	1
10OH	1-Decanol	Alcohols		1	1	0	0	1	1
12OH	1-Dodecanol	Alcohols		1	1	0	0	1	1
Et-4OH	2-Ethyl-1-butanol	Alcohols		1	1	0	0	1	1
Et-6OH	2-Ethyl-1-hexanol	Alcohols		1	1	0	0	1	1
2-3OH	2-Propanol	Alcohols		1	1	0	0	1	1
2-4OH	2-Butanol	Alcohols		1	1	0	0	1	1
2-5OH	2-Pentanol	Alcohols		1	1	0	0	1	1
2-7OH	2-Heptanol	Alcohols		1	1	0	0	1	1
3-7OH	3-Heptanol	Alcohols		1	1	0	0	1	1
2-10OH	2-Decanol	Alcohols		1	1	0	0	1	1
Cy6OH	Cyclohexanol	Alcohols		1	1	1	0 (F)	1	1
NH_2-2OH	Ethanolamine	Alcohols	Halogenated (F)	1	0 (F)	0	1 (F)	1	0 (F)
Cl-3OH	3-Chloro-1-propanol	Alcohols		1	1	0	0	1	1
C5	*n*-Pentane	Aliphatic		0	0	0	0	0	0
C6	*n*-Hexane	Aliphatic		0	0	0	0	0	0
C7	*n*-Heptane	Aliphatic		0	0	0	0	0	0
C8	*n*-Octane	Aliphatic		0	0	0	0	0	0
C9	*n*-Nonane	Aliphatic		0	0	0	0	0	0
C10	*n*-Decane	Aliphatic		0	0	0	1 (F)	0	0
C11	*n*-Undecane	Aliphatic		0	0	0	0	0	0
C12	*n*-Dodecane	Aliphatic		0	0	0	1	0	0
C8=	1-Octene	Aliphatic		0	0	0	0	0	0
C10=	1-Decene	Aliphatic		0	0	0	0	0	0
CyC5	Cyclopentane	Aliphatic		0	0	1	0 (F)	0	0
CyC6	Cyclohexane	Aliphatic		0	0	1	0 (F)	0	0
PhH	Benzene	Aromatic		0	0	1	1	0	0
PhMe	Toluene	Aromatic		0	0	1	1	0	0
PhEt	Ethylbenzene	Aromatic		0	0	1	1	0	0
o-Xy	1,2-Xylene	Aromatic		0	0	1	1	0	0
Mes	Mesitylene	Aromatic		0	0	1	1	0	0
C1C2	Methyl acetate	Esters		1	1	0	0	2	1 (F)
C1C3	Methyl propionate	Esters		1	1	0	0	2	2
C2C2	Ethyl acetate	Esters		1	1	0	0	2	2
C2C3	Ethyl propionate	Esters	Others (F)	1	0 (F)	0	0	2	0 (F)
C2C4	Ethyl butylate	Esters	Ethers (F)	1	1	0	0	2	2
C4C2	Butyl acetate	Esters	Halogenated (F)	1	1	0	0	2	2

Table S1. (*Continued*) Odor samples.

Label	Samples	Category		Oxygen (w/ or w/o)		Ring (w/ or w/o)		No. of Oxygen	
		True	Prediction	True	Prediction	True	Prediction	True	Prediction
2O2	Diethyl ether	Ethers	Esters (F)	1	1	0	0	1	2 (F)
6O6	Dihexyl ether	Ethers	Halogenated (F)	1	1	0	1 (F)	1	0 (F)
Cy5O	Tetrahydrofuran	Ethers		1	1	1	0 (F)	1	2 (F)
Cy6O	Tetrahydropyran	Ethers		1	1	1	1	1	2 (F)
Cy6O_2	1,4-Dioxiane	Ethers		1	1	1	1	2	1 (F)
Cy6ON	Morpholine	Ethers	Acids (F)	1	1	1	0 (F)	1	1
Cl_2-C1	Dichloromethane	Halogenated	Ethers (F)	0	1 (F)	0	0	0	1 (F)
Cl_3-C1	Chloroform	Halogenated		0	0	0	0	0	0
Cl_4-C1	Carbon tetrachloride	Halogenated		0	0	0	0	0	0
Cl_2-C2	1,2-Dichloroethane	Halogenated		0	0	0	0	0	0
Cl_4-C2	1,1,2,2-Tetrachloroethane	Halogenated		0	0	0	0	0	0
Cl-C5	1-Chloropentane	Halogenated		0	0	0	0	0	0
Cl-C6	1-Chlorohexane	Halogenated		0	0	0	0	0	0
Br_3-C1	Bromoform	Halogenated	Ethers (F)	0	1 (F)	0	0	0	1 (F)
Br_2-C2	1,2-Dibromoethane	Halogenated		0	0	0	0	0	0
Br_4-C2	1,1,2,2-Tetrabromoethane	Halogenated		0	1 (F)	0	0	0	1 (F)
Cl-CyC6	Chlorocyclohexane	Halogenated		0	0	1	1	0	0
R_f-CyC6	Perfluoromethylcyclohexane	Halogenated		0	1 (F)	1	1	0	0
PhF	Fluorobenzene	Halogenated		0	0	1	1	0	0
PhCl	Chlorobenzene	Halogenated		0	0	1	1	0	0
PhBr	Bromobenzene	Halogenated		0	0	1	1	0	0
PhI	Iodobenzene	Halogenated		0	0	1	1	0	0
o-ArF_2	1,2-Difluorobenzene	Halogenated		0	0	1	1	0	0
o-$ArCl_2$	1,2-Dichlorobenzene	Halogenated		0	0	1	1	0	0
m-$ArCl_2$	1,3-Dichlorobenzene	Halogenated		0	0	1	1	0	0
o-ArClBr	1-Bromo-2-chlorobenzene	Halogenated		0	0	1	1	0	0
o-$ArBr_2$	1,2-Dibromobenzene	Halogenated		0	0	1	1	0	0
ArF_6	Hexafluorobenzene	Halogenated		0	0	1	1	0	0
1C=O1	Acetone	Ketones		1	1	0	0	1	1
1C=O2	Methyl ethyl ketone	Ketones		1	1	0	0	1	1
1C=O4	Methyl butyl ketone	Ketones		1	1	0	1 (F)	1	1
2C=O2	Diethyl ketone	Ketones		1	1	0	0	1	1
2C=O4	Ethyl butyl ketone	Ketones		1	1	0	0	1	1
3C=O3	Dipropyl ketone	Ketones		1	1	0	0	1	1
CyC5C=O	Cyclopentanone	Ketones		1	0 (F)	1	0 (F)	1	1
CyC6C=O	Cyclohexanone	Ketones		1	0 (F)	1	1	1	0 (F)
H_2O	Water	Others	Alcohols (F)	1	1	0	0	1	1
6CHO	Hexanal	Others	Ketones (F)	1	1	0	1 (F)	1	1
PhCHO	Benzaldehyde	Others		1	1	1	1	1	2 (F)
2CN	Acetonitrile	Others	Alcohols (F)	0	1 (F)	0	0	0	1 (F)
PhCN	Benzonitrile	Others		0	1 (F)	1	1	0	1 (F)
DMF	*N,N*-dimethylformamide	Others	Alcohols (F)	1	1	0	0	1	1
1S1	Dimethyl sulfide	Others	Halogenated (F)	0	0	0	0	0	2 (F)

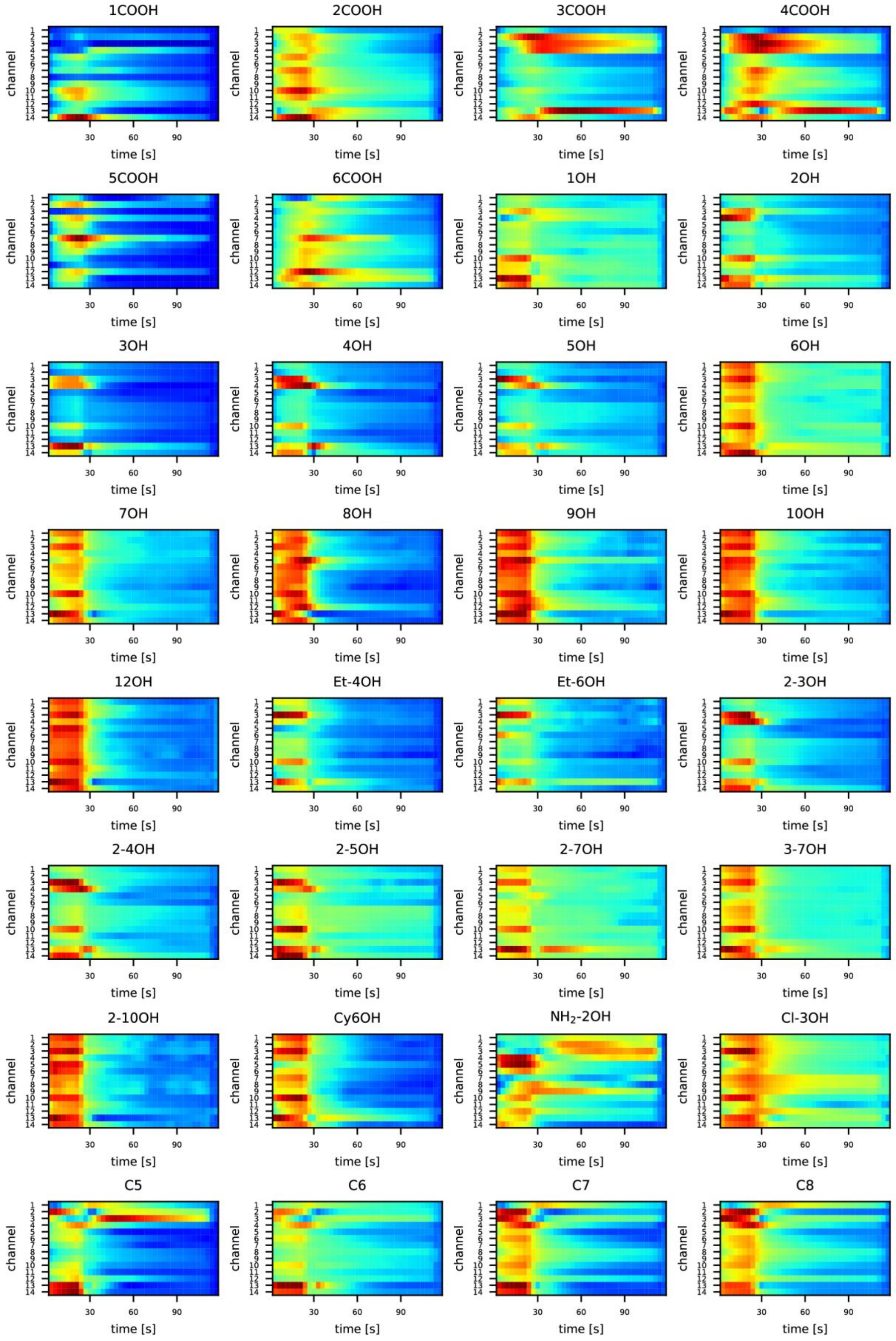

Figure S1. Importance maps for all the samples when the category classification was performed.

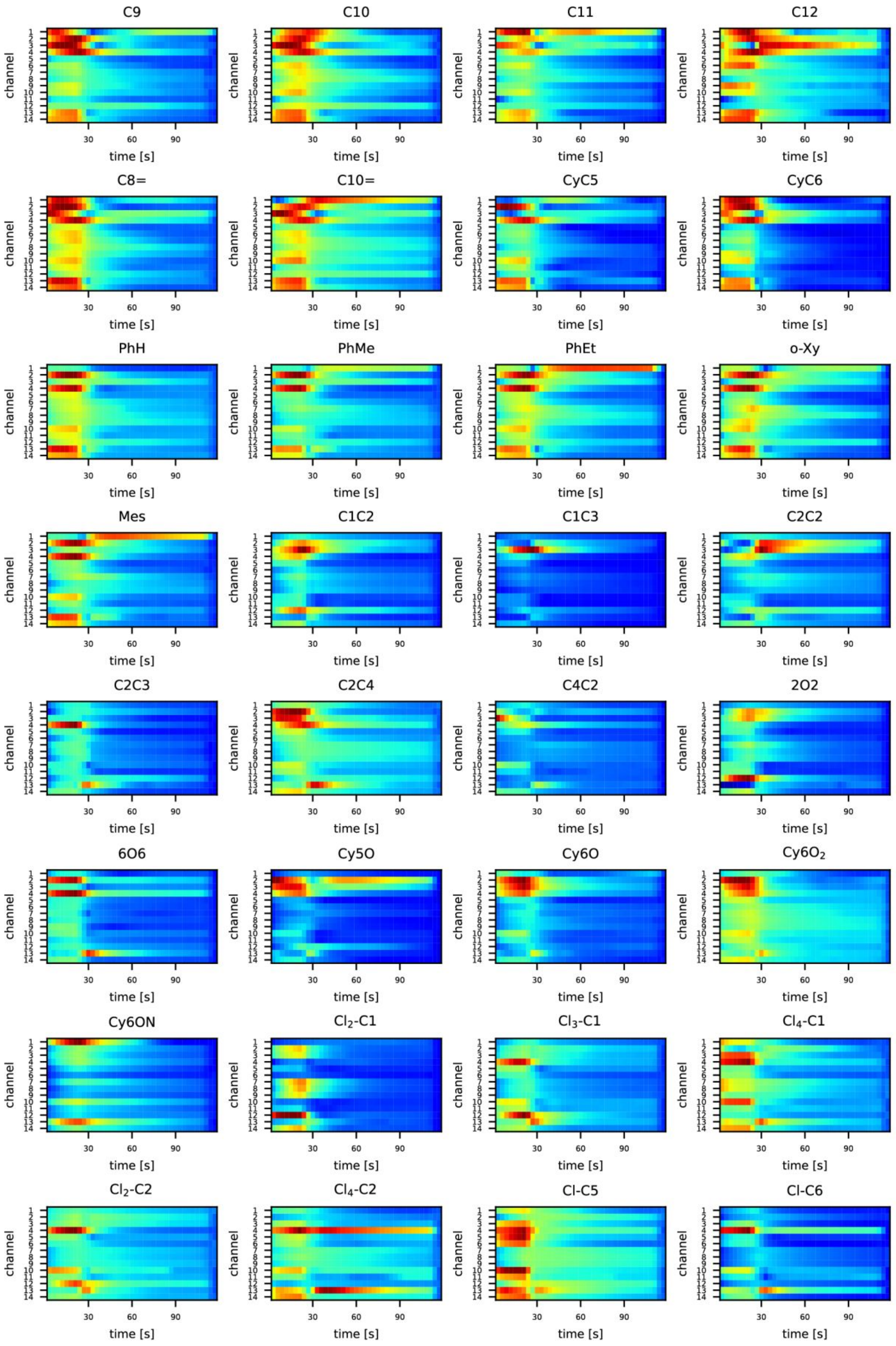

Figure S1. (*Continued*) Importance maps for all the samples when the category classification was performed.

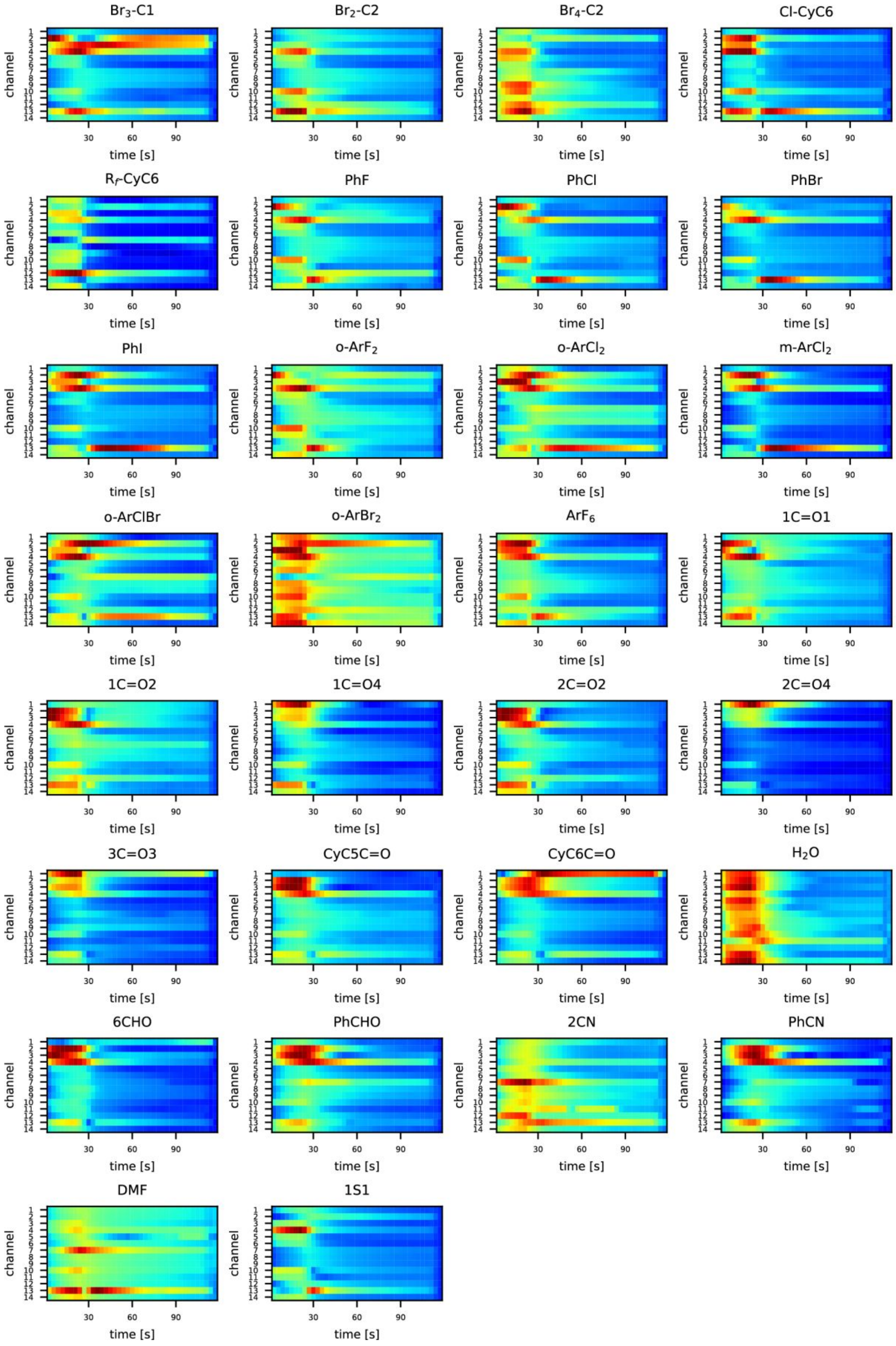

Figure S1. (*Continued*) Importance maps for all the samples when the category classification was performed.

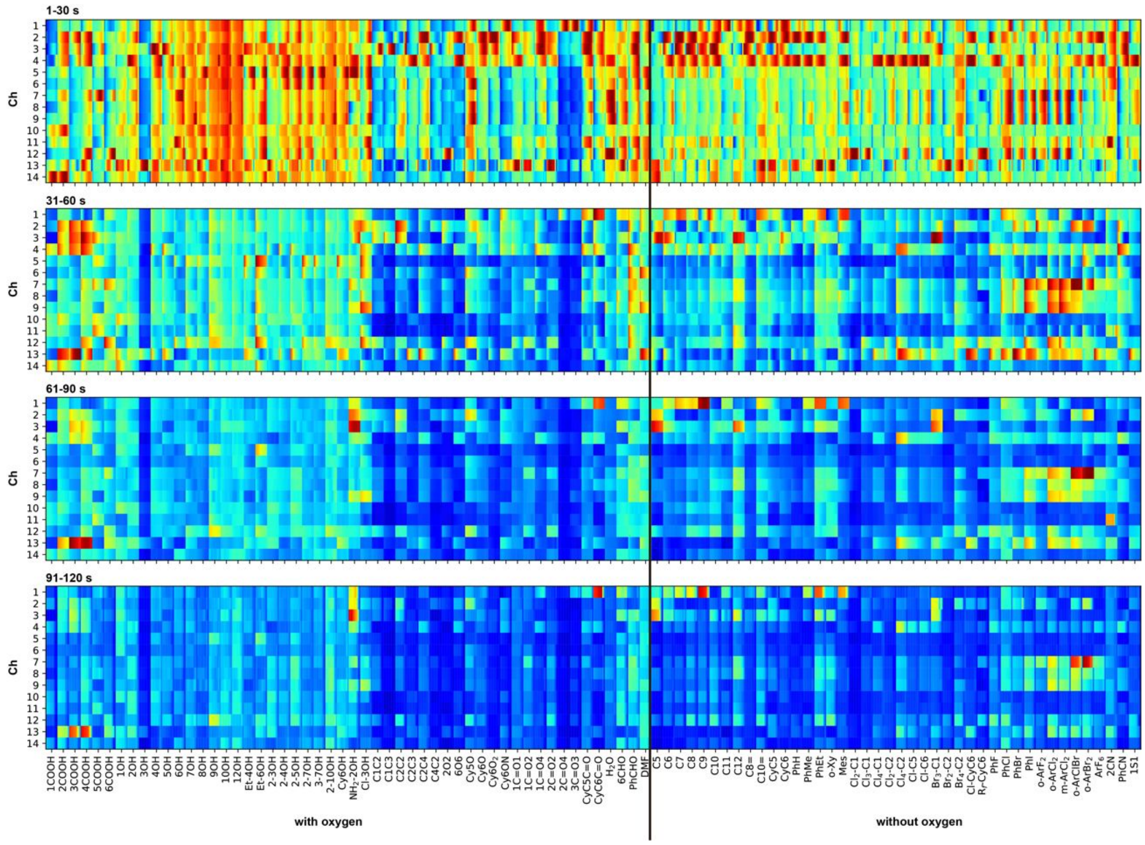

Figure S2. Importance maps generated using the Score-CAM method when the existence of oxygen atom is predicted.

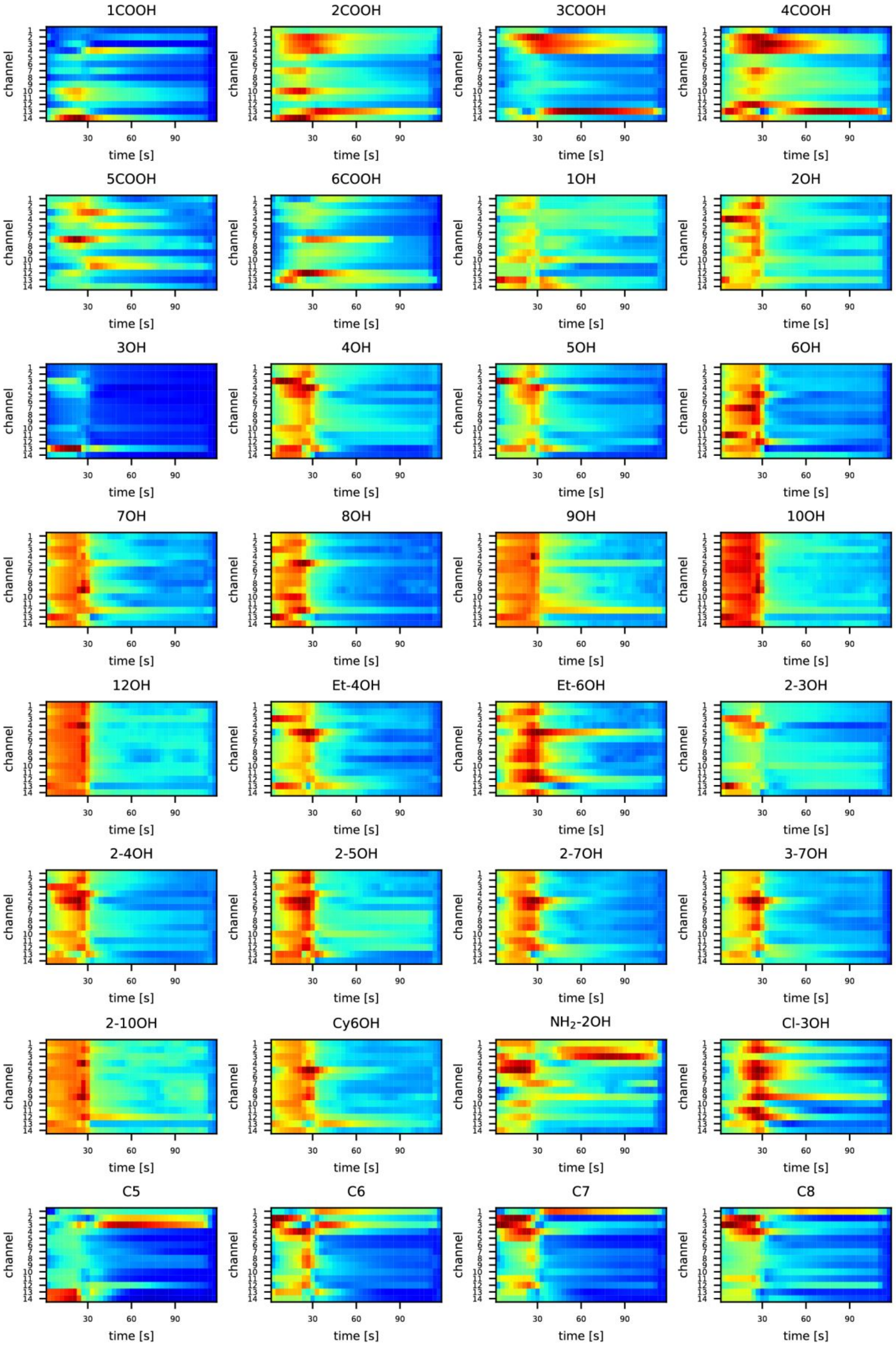

Figure S3. Importance maps for all the samples when the oxygen atoms classification was performed.

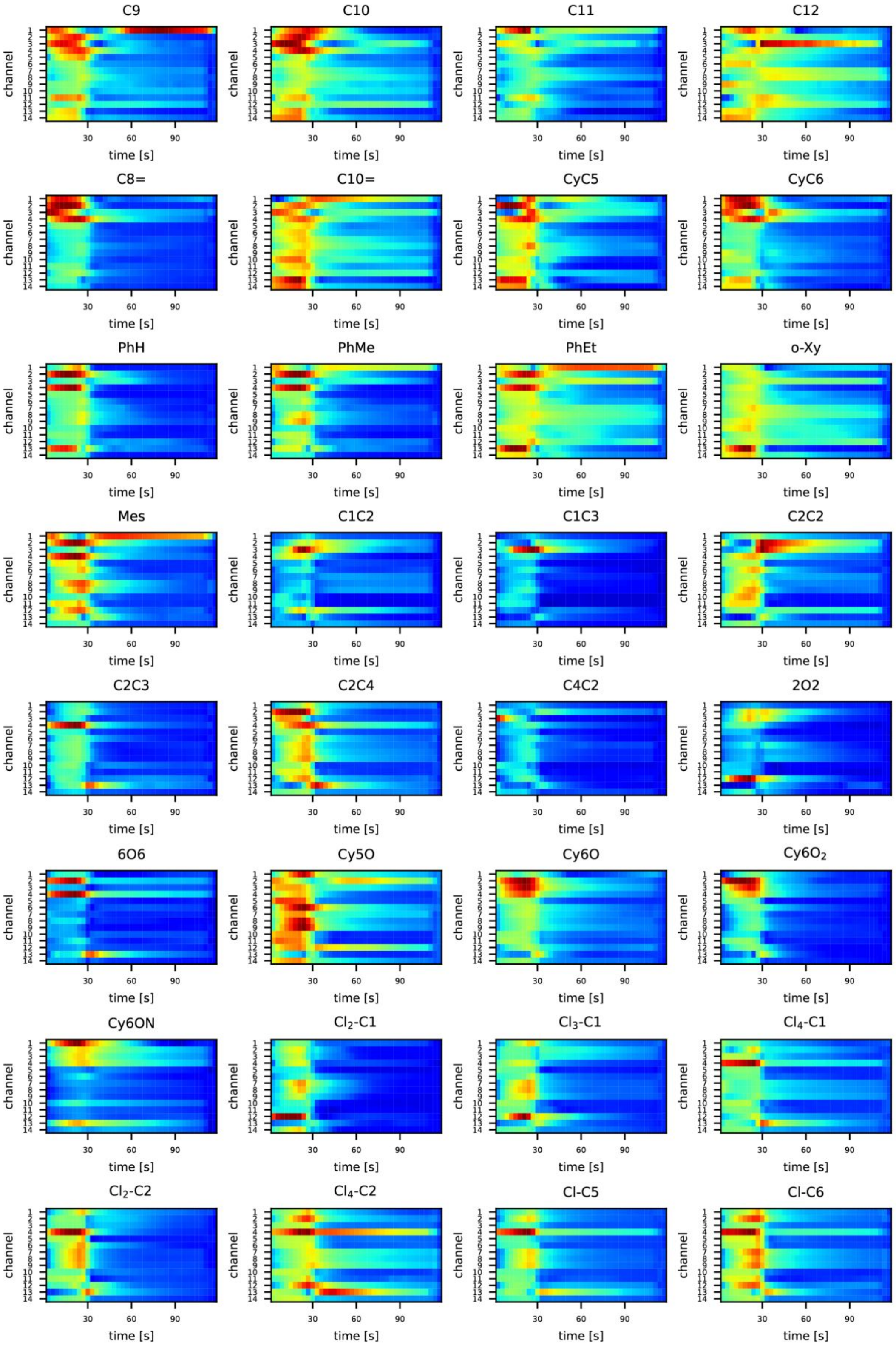

Figure S3. (*Continued*) Importance maps for all the samples when the oxygen atoms classification was performed.

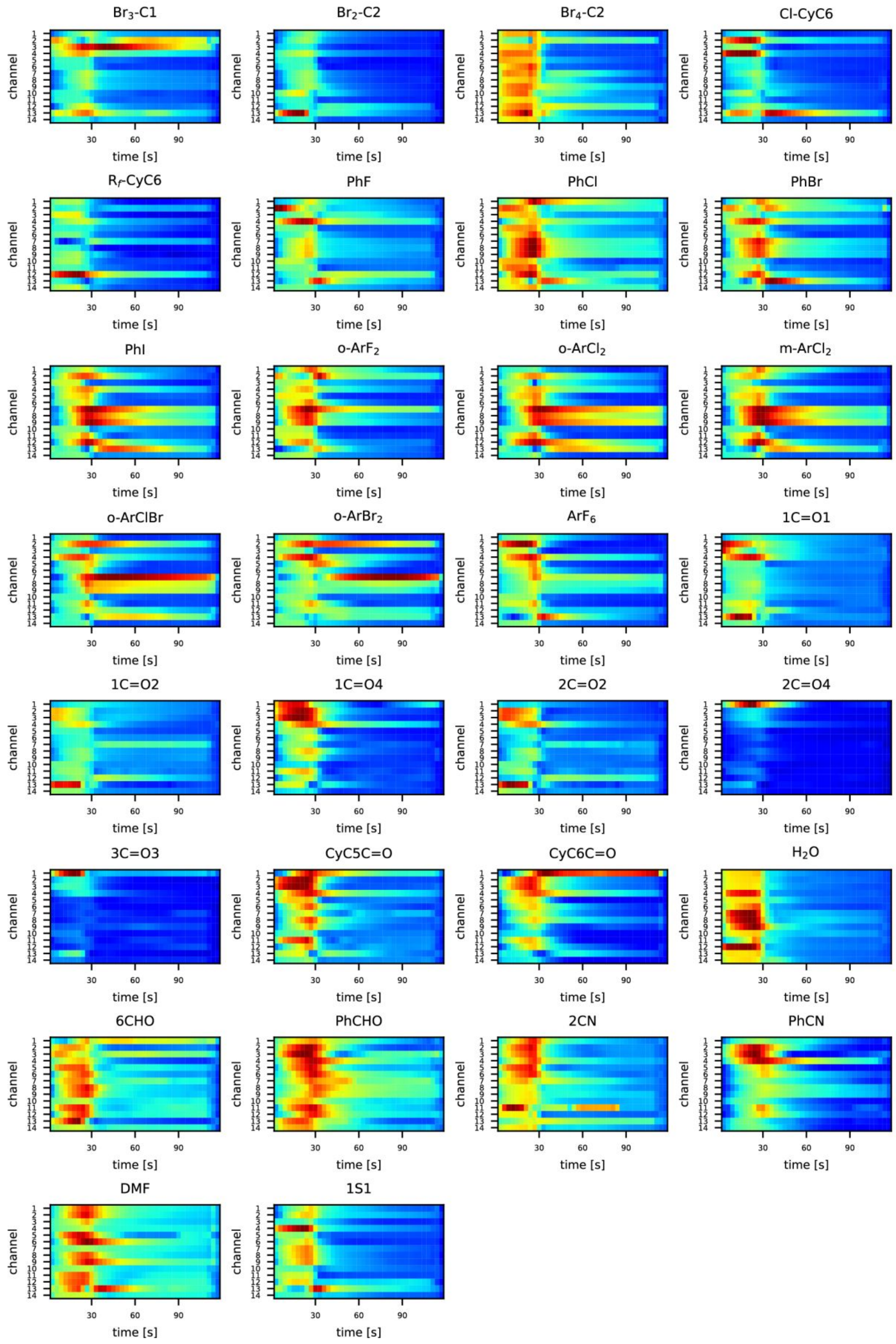

Figure S3. (*Continued*) Importance maps for all the samples when the oxygen atoms classification was performed.

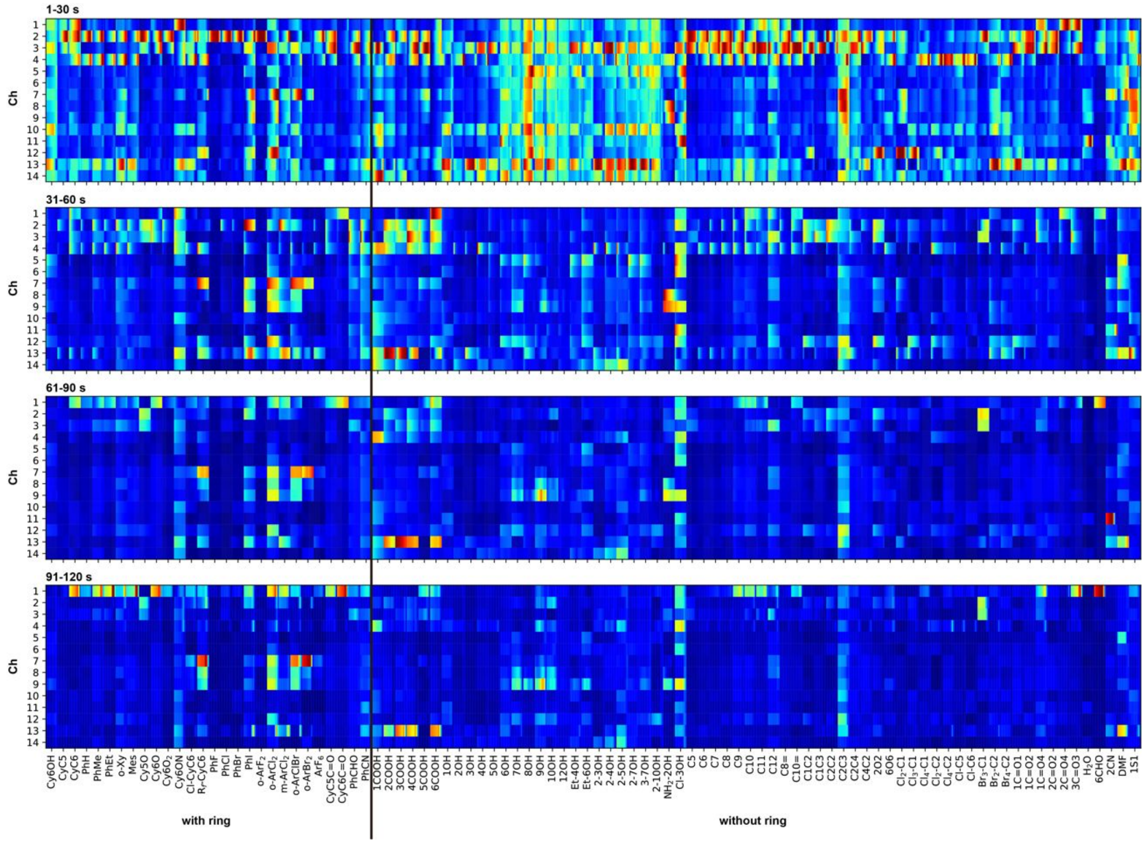

Figure S4. Importance maps generated using the Score-CAM method when the existence of ring structure is predicted.

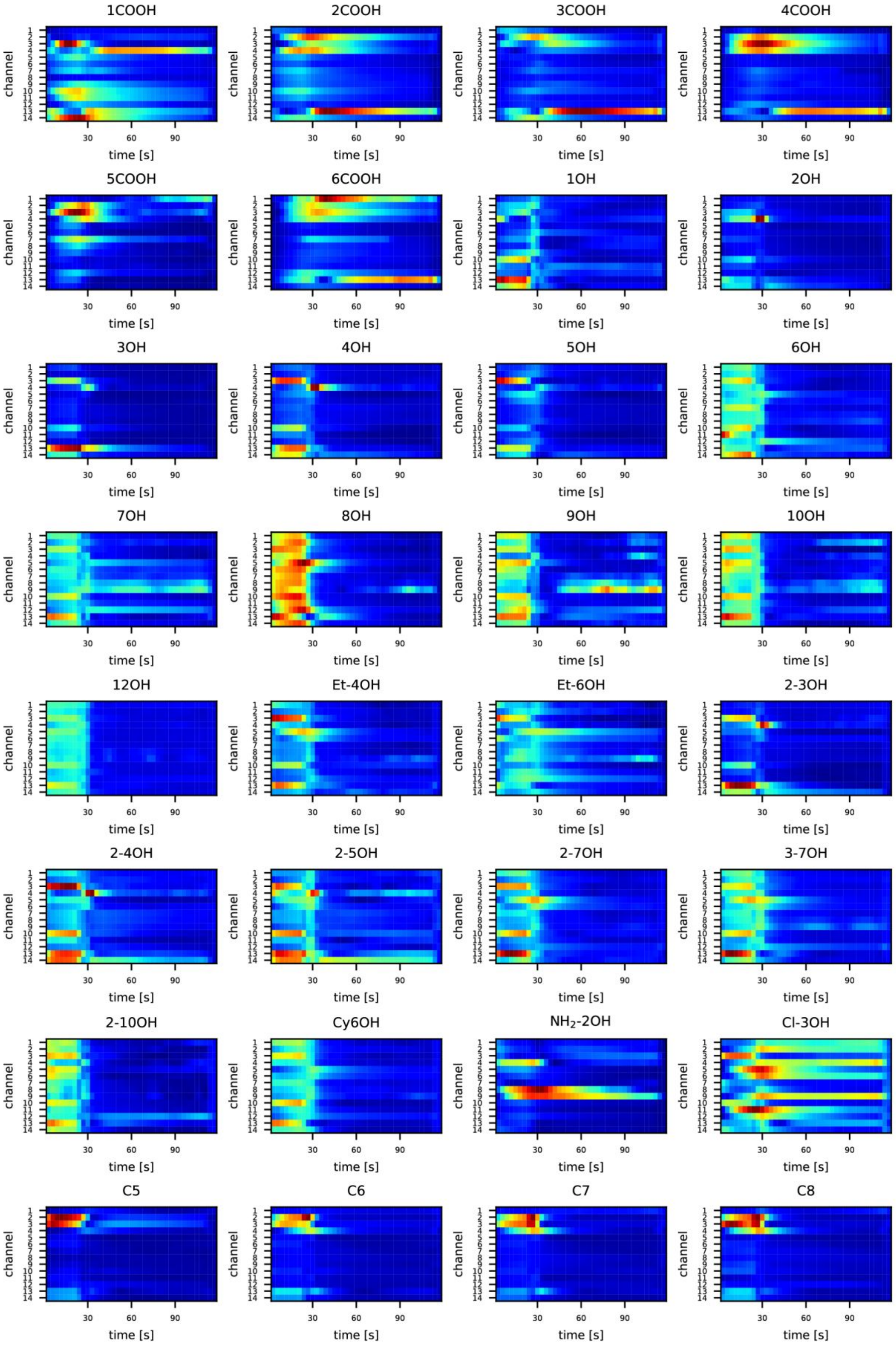

Figure S5. Importance maps for all the samples when the ring structures classification was performed.

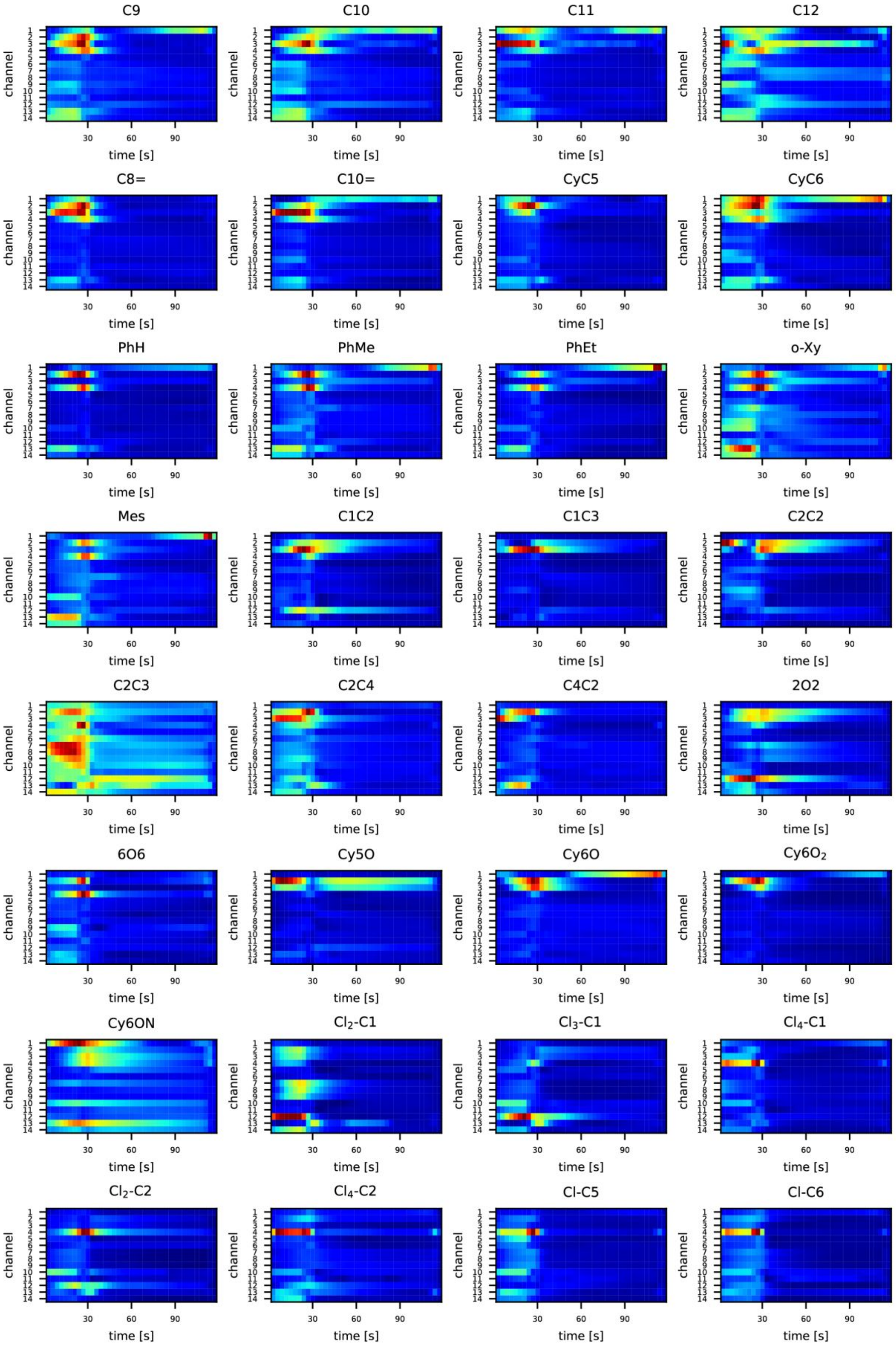

Figure S5. (*Continued*) Importance maps for all the samples when the ring structures classification was performed.

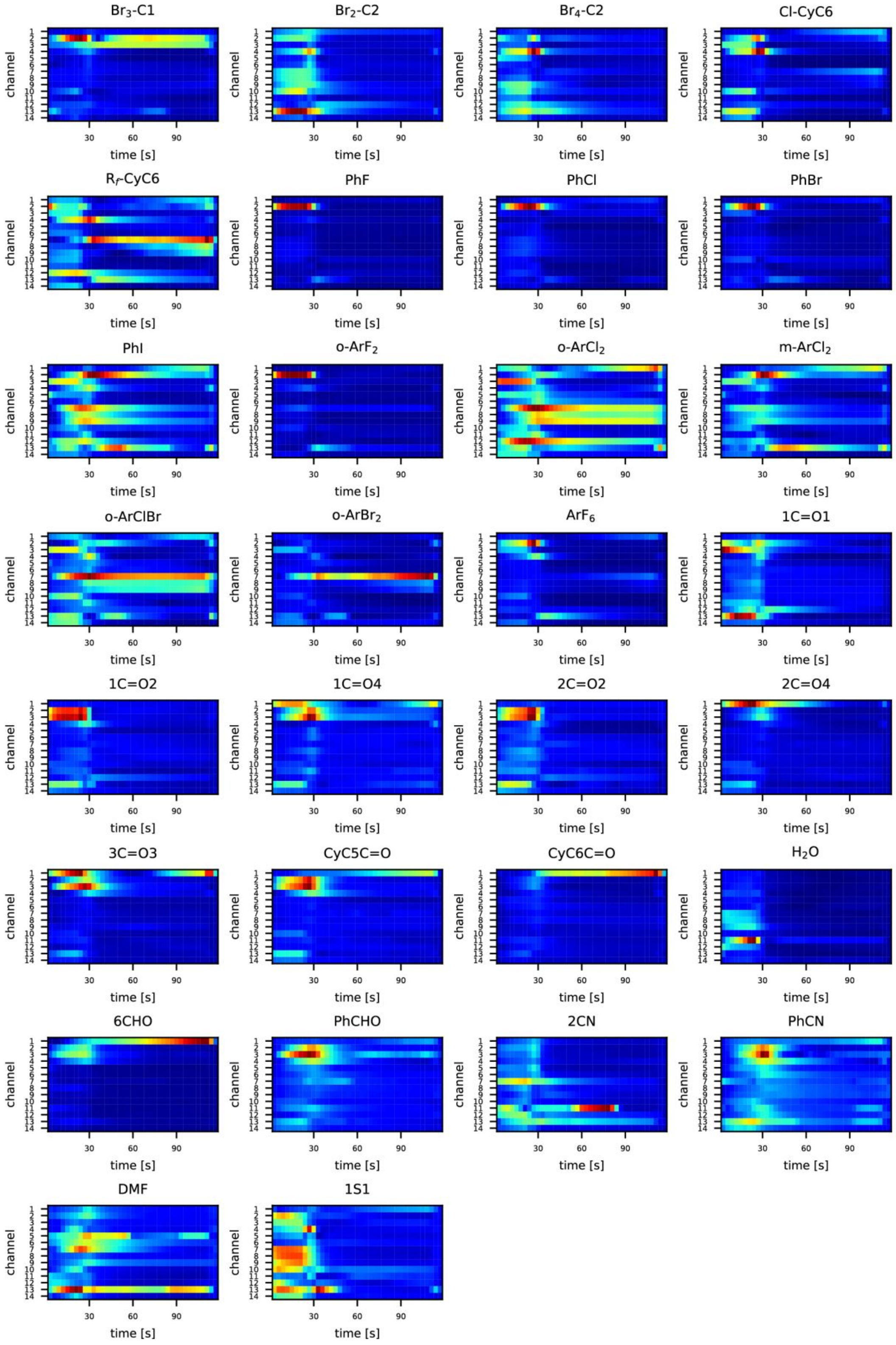

Figure S5. (*Continued*) Importance maps for all the samples when the ring structures classification was performed.

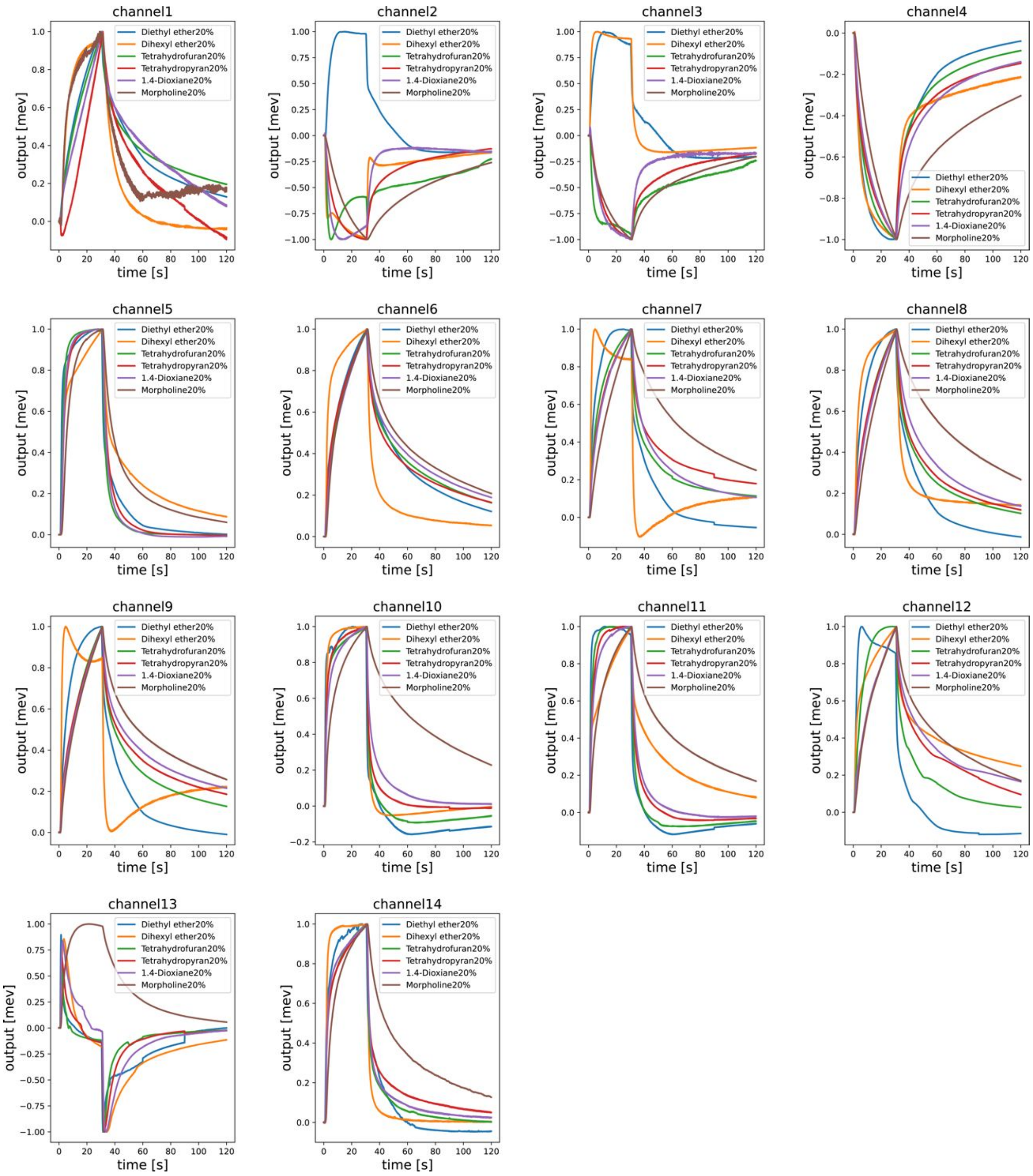

Figure S6. MSS signals of ethers at a concentration of 20%.

Supporting Note A: Definition of the four-dimensional features of the signals

In this note, we showed the four-dimensional features generated by focusing on the importance of signals, such as adsorption or desorption processes. Examples of MSS signals are shown in Figure S7, and five values, denoted as a, b, c, d, and e, were extracted. Here, $t_a = 0$, $t_b = 2.5$, $t_c = 30$, and $t_d = 32.5$ were used, and the value of e represents the maximum absolute value of the signals. From these values, we defined four parameters as follows: parameter 1: $b - a$, parameter 2: $c - b$, parameter 3: $d - c$, and parameter 4: $e - a$. For each channel, these four parameters were obtained and used as features to train SVM and RF models.

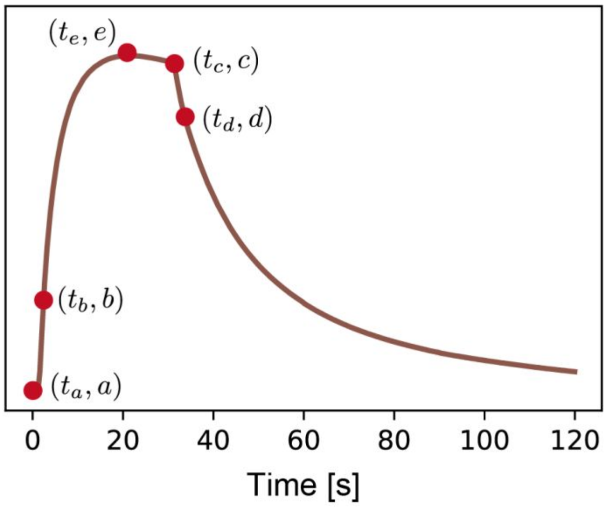

Figure S7. Five values of a, b, c, d, and e extracted from a signal.
